# The prognostic impact of tumor location in nonmuscle-invasive bladder cancer patients undergoing transurethral resection: insights from a cohort study utilizing Chinese multicenter and SEER registries

**DOI:** 10.1097/JS9.0000000000001675

**Published:** 2024-05-24

**Authors:** Lilong Liu, Kaiwen Li, Shao-gang Wang, Chinese Bladder Cancer Consortium, Jianli Wang, Zhipeng Yao, Yu Xie, Zhigang Ji, Zhiwen Chen, Hailong Hu, Haige Chen, Junyi Hu, Yaxin Hou, Zhenghao Liu, Yang Li, Yuhong Ding, Yingchun Kuang, Yang Xun, Jia Hu, Jiaqiao Zhang, Heng Li, Tie Chong, Jianbin Bi, Zhiping Wang, Yinhuai Wang, Peng Zhang, Qiang Wei, Zhaohui Chen, Lei Li, Jian Huang, Zheng Liu, Ke Chen

**Affiliations:** a Department of Urology, Tongji Hospital, Tongji Medical College, Huazhong University of Science and Technology, Wuhan; b Department of Urology, Sun Yat-Sen Memorial Hospital, Sun Yat-Sen (Zhongshan) University, Guangzhou, Guangdong; c Department of Urology, Hubei Cancer Hospital, Tongji Medical College, Huazhong University of Science and Technology, Wuhan, Hubei; d Department of Urology, The Affiliated Cancer Hospital of Xiangya School of Medicine of Central South University, Hunan Cancer Hospital, Changsha, Hunan; e Department of Urology, Peking Union Medical College Hospital, Chinese Academy of Medical Sciences and Peking Union Medical College, Beijing; f Department of Urology, The First Affiliated Hospital of the Third Military Medical University, Chongqing; g Department of Urology, The Second Hospital of Tianjin Medical University, Tianjin; h Department of Urology, Renji Hospital Affiliated to Shanghai Jiao Tong University School of Medicine, Shanghai; i Department of Urology, The Second Affiliated Hospital of Xi’an Jiaotong University, Xi’an, Shanxi; j Department of Urology, The First Hospital of China Medical University, Shenyang, Liaoning; k Clinical Center of Gansu Province for Urological Diseases, Lanzhou University Second Hospital, Lanzhou City, Gansu; l Department of Urology, The Second Xiangya Hospital, Central South University, Changsha, Hunan; m Department of Urology, West China Hospital, Sichuan University, Chengdu, Sichuan; n Department of Urology, Union Hospital, Tongji Medical College, Huazhong University of Science and Technology, Wuhan, Hubei; o Department of Urology, The First Affiliated Hospital of Xi’an Jiaotong University, Xi’an, Shanxi, People’s Republic of China

**Keywords:** bladder dome, nonmuscle invasive bladder cancer, partial cystectomy, transurethral resection of bladder tumors, tumor location

## Abstract

**Objective:**

Most bladder cancers are nonmuscle invasive bladder cancer (NMIBC), and transurethral resection of bladder tumors (TURBT) is the standard treatment. However, postoperative recurrence remains a significant challenge, and the influence of bladder tumor location on prognosis is still unclear. This study aims to investigate how tumor location affects the prognosis of NMIBC patients undergoing TURBT and to identify the optimal surgical approach.

**Methods:**

A multicenter study was conducted, which included Chinese NMIBC data from 15 hospitals (1996–2019) and data from 17 registries of the Surveillance, Epidemiology, and End Results database (SEER) (2000–2020). Patients initially diagnosed with NMIBC and undergoing TURBT or partial cystectomy were analyzed, with cases lost to follow-up or with missing data excluded. The study investigated the overall survival (OS), disease-specific survival (DSS), and recurrence-free survival (RFS) among patients with different tumor locations. Kaplan–Meier, Cox regression, and propensity score matching methods were employed to explore the association between tumor location and prognosis. Stratified populations were analyzed to minimize bias.

**Results:**

This study included 118 477 NMIBC patients and highlighted tumor location as a crucial factor impacting post-TURBT prognosis. Both anterior wall and dome tumors independently predicted adverse outcomes in two cohorts. For anterior wall tumors, the Chinese cohort showed hazard ratios (HR) for OS of 4.35 (*P*<0.0001); RFS of 2.21 (*P*<0.0001); SEER cohort OS HR of 1.10 (*P*=0.0001); DSS HR of 1.13 (*P*=0.0183). Dome tumors displayed similar trends [Chinese NMIBC cohort OS HR of 7.91 (*P*<0.0001); RFS HR of 2.12 (*P*<0.0001); SEER OS HR of 1.05 (*P*=0.0087); DSS HR of 1.14 (*P*=0.0006)]. Partial cystectomy significantly improved the survival of dome tumor patients compared to standard TURBT treatment (*P*<0.01).

**Conclusion:**

This study reveals the significant impact of tumor location in NMIBC patients on the outcomes of TURBT treatment, with tumors in the anterior wall and bladder dome showing poor post-TURBT prognosis. Compared to TURBT treatment, partial cystectomy improves the prognosis for bladder dome tumors. This study provides guidance for personalized treatment and prognosis management for NMIBC patients.

## Introduction

Highlights

- Transurethral resection of bladder tumors (TURBT) is the standard treatment for nonmuscle invasive bladder cancer (NMIBC) patients. This study aims to investigate how tumor location (anterior wall, bladder neck, dome, lateral wall, posterior wall, trigone, urachus, ureteric orifice, and overlapping lesion) influences the prognosis of NMIBC patients after TURBT treatment and to identify optimal surgical approaches.
- It was revealed that tumors located in the anterior wall and dome independently predicted adverse outcomes in this multicenter study, which involved 118 477 NMIBC patients [5569 from Chinese cohort, 112 908 from the Surveillance, Epidemiology, and End Results database (SEER)]. Tumors located in anterior wall showed significant hazard ratios (HR) for Chinese cohort overall survival (OS) (HR 4.35), recurrence-free survival (RFS) (HR 2.21), and SEER OS (HR 1.10), disease-specific survival (DSS) (HR 1.13); while dome tumors displayed elevated HR for Chinese cohort OS (HR 7.91), RFS (HR 2.12), and SEER OS (HR 1.05), DSS (HR 1.14); additionally, partial cystectomy significantly improved the survival of patients with dome tumors compared to standard TURBT treatment (*P*<0.01).
- Tumor location significantly influences the outcomes of TURBT treatment in NMIBC patients, with tumors located in anterior wall and bladder dome possessing worse post-TURBT prognosis; partial cystectomy improves the prognosis of bladder dome tumors compared to TURBT.

Bladder cancer ranks 10th globally as the most common tumor, with over 573 000 new cases and 212 000 deaths in 2020^1^. It is the 6th most common cancer in men and the 17th in women. Nonmuscle invasive bladder cancer (NMIBC) constitutes 75% of all cases^2^, and transurethral resection of bladder tumors (TURBT) is the primary treatment, aiming for complete tumor removal, and essential diagnosis information^3^. However, the oncological control of NMIBC patients is far from satisfactory, with a 1-year recurrence rate of 15–61% and a 5-year recurrence rate of 31–78%^4^. The 10-year cancer-specific survival for high-grade NMIBC patients is 70–85%, while low-grade TA lesions progress at about 6%, and high-grade T1 lesions face an increased risk of progression of 17%^5^. Seeking mechanisms for postoperative tumor recurrence and progression is crucial for further improving the prognosis of bladder cancer patients.

**Figure 1:**
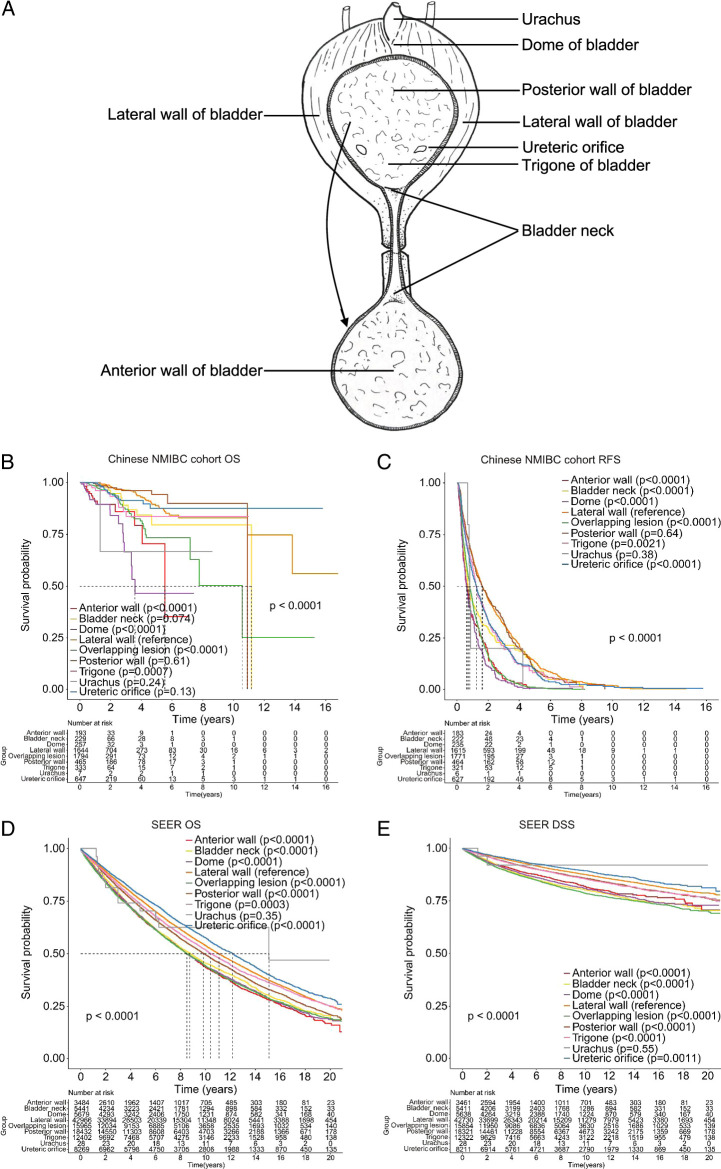
Prognosis of NMIBC patients with tumors in different locations was compared across all included individuals in the Chinese multicenter cohort and SEER database. (A) NMIBC is classified into nine types based on tumor location: anterior wall, bladder neck, dome, lateral wall, posterior wall, trigone, urachus, ureteric orifice, and overlapping lesion. Comparison of overall survival (B) and recurrence-free survival (C) among patients with tumors in different locations in the Chinese multicenter cohort. Comparison of overall survival (D) and disease-specific survival (E) among patients with tumors in different locations in the SEER database.

In this study, NMIBC patients were classified into nine types based on tumor location: anterior wall, bladder neck, dome, lateral wall, posterior wall, trigone, urachus, ureteric orifice, and overlapping lesion (Fig. 1A). Due to significant variation in tumor incidence (0.13–32.21%) among these locations, conducting prospective studies for prognostic comparisons is challenging. Limited research has explored how tumor location affects the post-TURBT prognosis of NMIBC patients^6–10^. However, there is currently no comprehensive study compares the prognosis across all nine locations, and major urology guidelines do not consider tumor location as a prognostic indicator for NMIBC patients after TURBT treatment. To address this gap, a large-scale international cohort study is essential for understanding the significance of tumor location in NMIBC treatment and prognosis. This study includes patients from the Chinese multicenter NMIBC cohort (Chinese NMIBC cohort) and the SEER database who underwent TURBT or partial cystectomy. The aim is to investigate the impact of tumor location on the postoperative prognosis of NMIBC patients undergoing TURBT treatment, and to explore the differences between various surgical approaches to seek the optimal surgical approaches.

## Methods

### Study design and participants

This study utilized data from the Chinese NMIBC cohort and the SEER 17 registries cohort, which was freely available to global researchers. In both cohorts, the inclusion criteria were limited to newly diagnosed NMIBC patients (Stage TA, TIS, and T1) who underwent either TURBT or partial cystectomy (SEER TURBT Code as 27 and partial cystectomy Code as 30). The Chinese NMIBC cohort included patients from January 1996 to December 2019 at 15 institutions. SEER*Stat software (version 8.4.1.1) collected 17 registries cohort data on NMIBC patients diagnosed between 2000 and 2020 (https://seer.cancer.gov/data-software/). The collected data included age, sex, race, residential location (rural-urban), income, year of diagnosis, tumor location, number of tumors, T stage, Grade, histologic type, chemotherapy, and patients’ prognostic information such as overall survival (OS), disease-specific survival time (DSS), and recurrence-free survival time (RFS). Tumor location information referred to the SEER Program Coding and Staging Manual 2023 (https://seer.cancer.gov/search?q=SEER+Program). Participants with unknown survival time or missing tumor location information were excluded (Supplementary Figure S1 and S2, Supplemental Digital Content 1, http://links.lww.com/JS9/C657). The study received approval from the Ethics Review Committee, and the requirement for informed consent was also waived because the retrospective data analysis posed no additional harm to the patients. The work has been reported in line with the strengthening the reporting of cohort, cross-sectional, and case–control studies in surgery (STROCSS) criteria^11,12^ (Supplemental Digital Content 2, http://links.lww.com/JS9/C658).

### Procedures and outcomes

Data from the Chinese NMIBC cohort were meticulously collected by trained personnel to minimize bias. Tumor locations included the anterior wall, bladder neck, dome, lateral wall, posterior wall, trigone, urachus, ureteric orifice, and overlapping lesions (Fig. 1A). Age was categorized as 18–64 or ≥65 years. T stage classification followed AJTT 3rd (1988–2003) or Derived AJTT T, 6th ed (2004–2015) or Derived SEER Combined T (2016–2017) or Derived EOD 2018 T (2018+). Grade was based on the 2004 WHO grading system in the Chinese NMIBC cohorts and Grade Recode (thru 2017) in the SEER database. Number of tumors was classified as single, multiple, or unknown. Race, rural-urban classification, income, and chemotherapy were categorized accordingly in the SEER database. Histologic types included urothelial carcinoma (TCC), adenocarcinoma (ACC), squamous carcinoma (SCC), or others. The primary outcome in both cohorts was OS, defined as the time from the first diagnosis to death or last follow-up. RFS was analyzed in the Chinese NMIBC cohort, and DSS was assessed in the SEER cohort. Survival analyses compared tumor location in all patients or within specific groups.

### Statistical analysis

The baseline characteristics across tumor locations were compared using the χ² test. Survival curves (OS, DSS, and RFS) were generated using Kaplan–Meier method, with differences assessed by log-rank trend test. To minimize biases, we conducted stratified survival analysis based on parameters such as patient age, sex, tumor stage, tumor grade, and tumor size, and performed univariate and multivariate Cox regression analyses, reporting hazard ratios (HR) with corresponding 95% CI. In the SEER cohort, a 1:1 matching using propensity score matching was conducted to compare TURBT with partial cystectomy for anterior wall or dome tumors. All tests were two-sided, and significance was set at *P*<0.05. Analyses were conducted using R statistical software (version 4.2.2).

## Results

The study included 118 477 patients: 5569 from the Chinese NMIBC cohort (1996–2019) and 112 908 (TURBT: 112 666 cases; partial cystectomy: 242 cases) from the SEER database (2000–2020) (Supplementary Figure S1 and S2, Supplemental Digital Content 1, http://links.lww.com/JS9/C657). The median follow-up time for the Chinese NMIBC cohort was 12.7 months, while for the SEER database it was 66.0 months. The distribution of bladder cancer patients across different locations varied between the two cohorts. Notably, the Chinese NMIBC cohort had a significantly higher proportion (32.21%) of patients with tumors in the overlapping lesion of the bladder compared to the SEER cohort (14.17%). The proportions of patients with tumors in the lateral wall, posterior wall, and bladder trigone were slightly lower in the Chinese NMIBC cohort. The SEER cohort had a higher proportion of patients aged 65 and above (71.87%) compared to the Chinese NMIBC cohort (49.51%). In the Chinese NMIBC cohort, males accounted for 79.85% and females accounted for 20.15%, comparable to the SEER cohort with 77.44 male and 22.56% female (Supplementary Table S1 and S2, Supplemental Digital Content 1, http://links.lww.com/JS9/C657).

The patients with tumor in the bladder lateral wall were identified as the reference category in survival analysis. In the Chinese NMIBC cohort, patients with tumors in the anterior wall, dome, and overlapping lesion had the poorest prognosis, while those with tumors in the lateral wall, ureteric orifice, and posterior wall had the most favorable prognosis (Fig. 1B). Tumors in the anterior wall, dome, or overlapping lesions were associated with higher recurrence risk, whereas lateral wall, posterior wall, and ureteric orifice tumors had the lowest recurrence risk (Fig. 1C). Similar findings were observed in the SEER cohort, with anterior wall, dome, neck, and overlapping lesion tumors having the poorest prognosis. Conversely, lateral wall and ureteric orifice tumors showed the most favorable prognosis (Fig. 1D). Similar trends were observed in DSS analysis (Fig. 1E). Demographic and clinical differences were noted between NMIBC patients in different locations in both cohorts, with those in the anterior wall or dome having a higher proportion of individuals aged 65 or older, higher T stage, and higher tumor grade. The reverse trend was observed for TA stage and low tumor grade (Supplementary Table S1 and S2, Supplemental Digital Content 1, http://links.lww.com/JS9/C657).

Subsequently, the survival analyses were conducted by stratifying patients based on age (18–64, 65+), sex, T stage (TA, TIS, and T1), tumor grade (Chinese NMIBC cohort: Grade-PUNLMP, Grade I & II (Grade-low), Grade III and IV (Grade-high); SEER: Grade-low, Grade-high), number of tumors (single tumor, multiple tumors), or tumor size (≤20 mm, >20 mm) in both the Chinese NMIBC cohort and SEER cohort. In different subgroup survival analyses, similar results were obtained. Specifically, patients with tumors located in the bladder lateral wall or the ureteral orifice exhibited the best OS, DSS, or RFS. Conversely, patients with tumors in the bladder anterior wall, bladder dome, or overlapping lesion of bladder showed poorer prognosis (Supplementary Figure S3–S13, Supplemental Digital Content 1, http://links.lww.com/JS9/C657).

**Figure 2:**
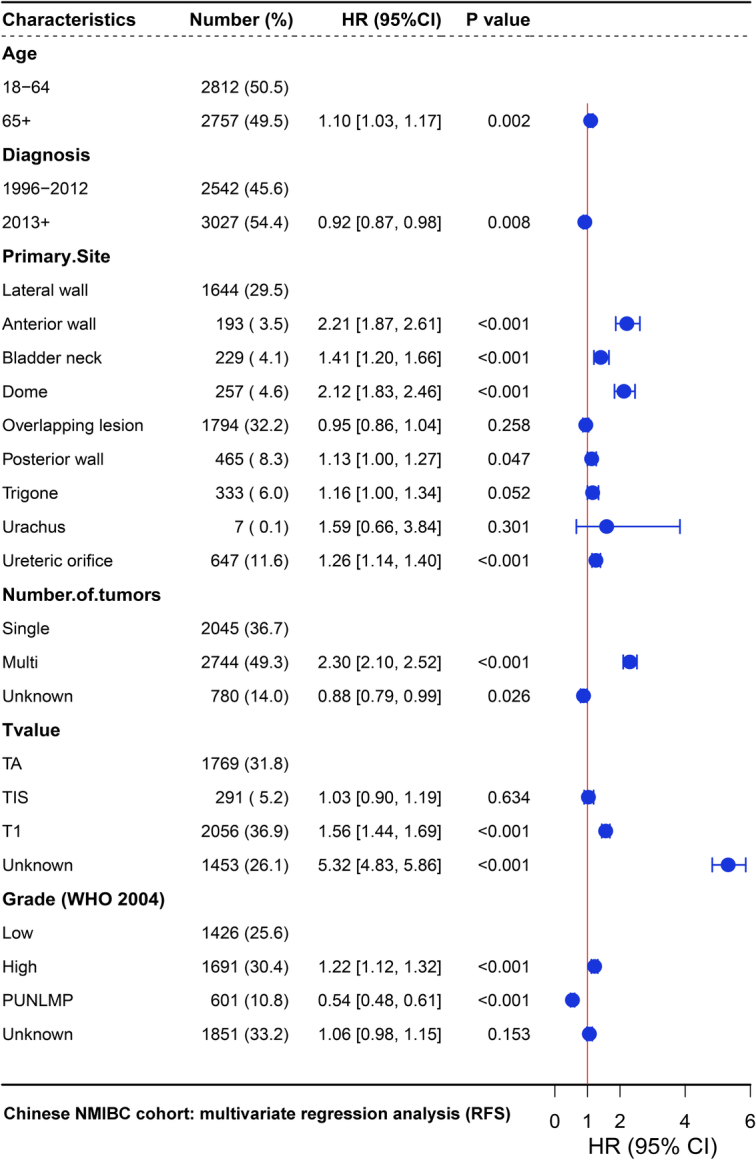
Multivariate COX analysis of the recurrence-free survival of NMIBC patients in the Chinese NMIBC cohort. ACC, adenocarcinoma; HR, hazard ratios; SCC, squamous carcinoma; TCC, urothelial carcinoma. The *P*-values represent differences among patients with tumors in different locations, and a *P*-value <0.05 indicates significant differences.

**Figure 3:**
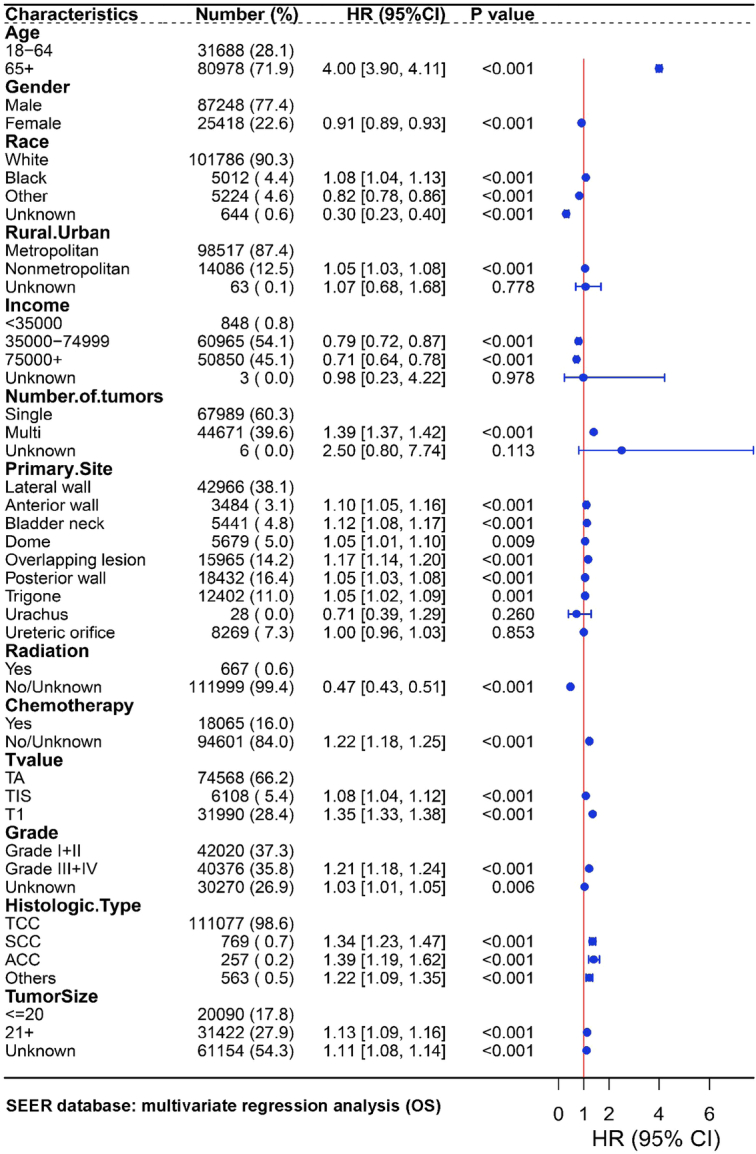
Multivariate COX analysis of the overall survival of NMIBC patients in the SEER database. The T stage in the SEER database was based on AJTT 3rd (1988–2003) or Derived AJTT T, 6th ed (2004–2015) or Derived SEER Combined T (2016–2017) or Derived EOD 2018 T (2018+). Grade was graded by Grade Recode (thru 2017) in the SEER database. ACC, adenocarcinoma; HR, hazard ratios; SCC, squamous carcinoma; TCC, urothelial carcinoma. The *P*-values represent differences among patients with tumors in different locations, and a *P*-value <0.05 indicates significant differences.

Through univariate and multivariate regression analyses of OS and RFS in the Chinese NMIBC cohort, it was found that patient age, tumor located in the bladder anterior wall or dome, number of tumors, T stage, and tumor grade were independent risk factors for patient recurrence and poor OS. Following univariate regression analysis, subsequent multivariate regression analysis identified patients with tumors located in the bladder anterior wall or dome had worse prognosis compared to patients with tumors in the bladder lateral wall: bladder anterior wall [RFS HR 2.21 (95% CI: 1.87–2.61), *P*<0.0001; OS HR 4.35 (95% CI: 2.44–7.77), *P*<0.0001]; bladder dome [RFS HR 2.12 (95% CI: 1.83–2.46), *P*<0.0001; OS HR 7.91 (95% CI: 4.84–12.94), *P*<0.0001; Supplementary Figure S14, Supplemental Digital Content 1, http://links.lww.com/JS9/C657, Fig. 2, and Supplementary Table S3, Supplemental Digital Content 1, http://links.lww.com/JS9/C657]. As for the SEER cohort, we found that patient age, Black ethnicity, residing in nonmetropolitan, tumor location outside of the bladder lateral wall or ureteral orifice, number of tumors, T stage, tumor grade, nonurothelial histology, and tumor size were independent prognostic risk factors. Multivariable Cox regression analysis found that patients with tumors located in the bladder anterior wall, bladder neck, bladder dome, overlapping lesion of bladder, and trigone had worse prognosis compared to patients with tumors in the lateral wall of bladder: bladder anterior wall [OS HR 1.10 (95% CI: 1.05–1.16), *P*=0.0001; DSS HR 1.13 (95% CI: 1.02–1.24), *P*=0.0183]; bladder neck [OS HR 1.12 (95% CI: 1.08–1.17), *P*<0.0001; DSS HR 1.39 (95% CI: 1.28–1.50), *P*<0.0001]; bladder dome [OS HR 1.05 (95% CI: 1.01–1.10), *P*=0.0087; DSS HR 1.14 (95% CI: 1.06–1.24), *P*=0.0006]; overlapping lesion of bladder [OS HR 1.17 (95% CI: 1.14–1.20), *P*<0.0001; DSS HR 1.43 (95% CI: 1.36–1.51), *P*<0.0001]; trigone: [OS HR 1.05 (95% CI: 1.02–1.09), *P*=0.0007; DSS HR 1.18 (95% CI: 1.11–1.26), *P*<0.0001] (Supplementary Figure S15, Supplemental Digital Content 1, http://links.lww.com/JS9/C657, Fig. 3 and Supplementary Table S4, Supplemental Digital Content 1, http://links.lww.com/JS9/C657). Taken together, the results suggested that tumors located in the bladder anterior wall and dome were associated with an unfavorable prognosis among NMIBC patients undergoing TURBT treatment.

**Figure 4:**
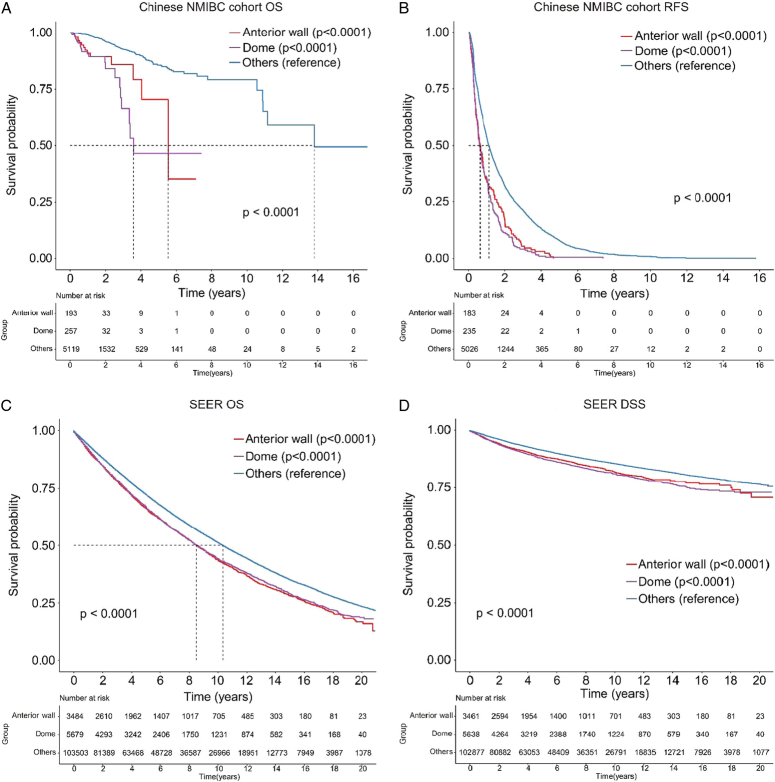
Comparing NMIBC patients’ prognosis with tumors in the bladder’s anterior wall or dome versus other locations was studied in the Chinese multicenter cohort and SEER database. Comparison of overall survival (A) and recurrence-free survival (B) among patients in the Chinese multicenter cohort. Comparison of overall survival (C) and disease-specific survival (D) among patients in the SEER database.

**Figure 5:**
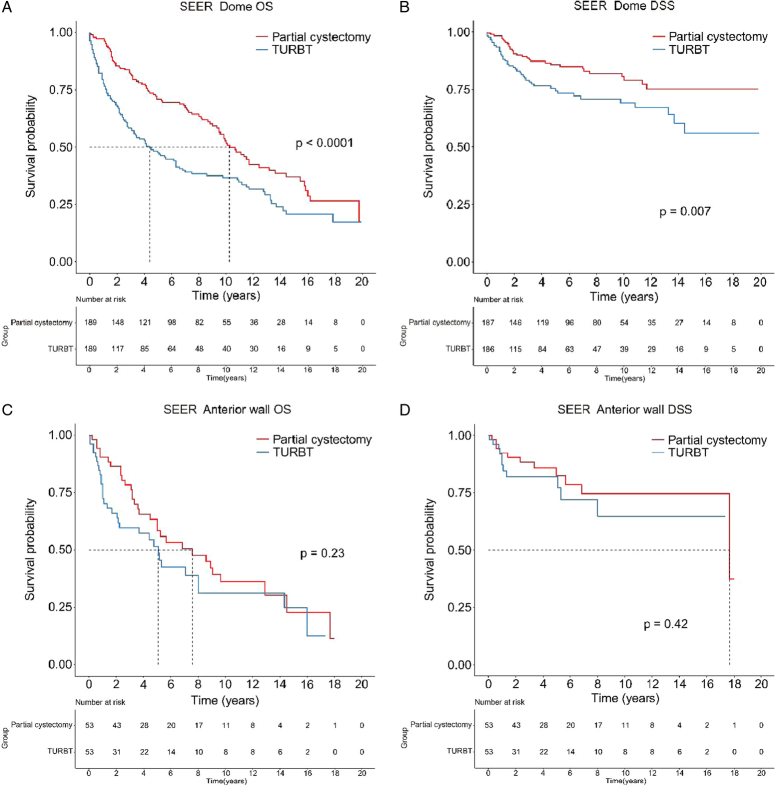
Comparing the prognosis of NMIBC patients with tumors in the bladder anterior wall and dome treated with partial cystectomy versus TURBT. Comparison of overall survival (A) and disease-specific survival (B) among patients with tumors in the dome; Comparison of overall survival (C) and disease-specific survival (D) among patients with tumors in the bladder anterior wall.

To determine if tumors in the bladder anterior wall and dome were risk factors for unfavorable prognosis in NMIBC patients after TURBT, we compared them to tumors in other locations. In both the Chinese NMIBC and SEER cohorts, patients with anterior wall or dome tumors had significantly worse prognosis than those in other locations (*P*<0.0001; Fig. 4A–D). Lastly, through using a 1:1 matching approach to compare TURBT with partial cystectomy (Supplementary Table S5, Supplemental Digital Content 1, http://links.lww.com/JS9/C657 and S6, Supplemental Digital Content 1, http://links.lww.com/JS9/C657), we found that partial cystectomy significantly improved the survival rate for dome tumors but not for anterior wall tumors, suggesting it might be a preferred surgical option for certain cases (Fig. 5A–D).

## Discussions

The EAU, AUA, and CUA guidelines for urological diseases do not currently incorporate tumor location as a prognostic factor for NMIBC patients undergoing TURBT. This multicenter study conducted in China and the US revealed that tumors in the bladder anterior wall or dome increase the risk of recurrence after TURBT treatment in NMIBC patients, affecting OS and DSS. Compared to TURBT, partial cystectomy improves the prognosis for bladder dome tumors. This finding advances our understanding of NMIBC patients, potentially influencing clinical practice by assisting clinicians in personalized treatment and prognosis assessment. It may also promote guideline revisions to incorporate tumor location as a prognostic indicator for NMIBC patients.

The incidence of bladder tumors varies widely, with only 3% of NMIBC patients having tumors in the anterior wall. Categorizing NMIBC patients into nine groups based on tumor location for prognostic comparisons is highly challenging due to the low incidence in some groups. There is limited publicly available research on how tumor location affects post-TURBT outcomes for NMIBC patients, and existing research findings are inconsistent. Most studies associate tumors in or involving the bladder neck with unfavorable post-TURBT outcomes^7–10,13^. Additionally, some studies suggest that bladder tumors in the posterior or lateral walls may be linked to early recurrence^6,7^. However, there is currently no reported comprehensive study comparing the prognosis of NMIBC patients across nine different locations^14^. This study, which includes over 110 000 patients from China and the USA, reveals variations in the results of univariate and multivariable Cox regression analyses between Chinese NMIBC data and the SEER database. These differences could be attributed to factors such as racial disparities, variations in treatment guidelines followed by surgeons, and discrepancies in patients’ living environments. Further research is needed to elucidate these differences. Nevertheless, both Chinese NMIBC data and SEER database data consistently highlight a notable connection between anterior wall and bladder dome tumors and unfavorable outcomes in NMIBC patients after TURBT. Overcoming past limitations, this concise yet comprehensive study offers valuable insights for future clinical research and personalized NMIBC diagnosis and treatment.

This study observed that patients with tumors located in the bladder’s anterior wall and dome tended to be older and had higher tumor grades. In contrast, those with tumors in the lateral wall and ureteric orifices were younger and had lower tumor grades, which was consistent with previous research findings^15^. The proposed explanation was that tumors in the ureteric orifices or lateral wall were more likely to cause visible hematuria early, facilitating early diagnosis. However, further research is necessary to validate this hypothesis. Despite the older age and higher tumor grades observed in patients with tumors in the anterior and dome, most survival analyses consistently indicated poorer prognoses for these patients compared to those with tumors in the lateral wall.

The bladder exhibits diverse microvascular networks across its different locations^16^, which may potentially influence the invasion and metastasis of localized bladder tumors (NMIBC and MIBC). Studies have indicated associations between tumor locations and factors like lymph node involvement and tumor stages. For instance, tumors located in the trigone or bladder neck have been associated with lymph node involvement^17^, while tumors in the bladder dome have been linked to higher tumor stages^15,17^. Another study found that tumors on the lateral wall or involving it were strongly associated with lymph node-positive disease, while tumors on the posterior bladder wall showed a lower tendency for lymph node metastasis^18^. However, reports also suggest that there are no significant differences in lymph node metastasis and RFS among bladder cancer patients with tumors in different locations undergoing radical cystectomy^19^. Whether tumors of NMIBC patients in the anterior and dome are more prone to muscle invasion or lymphatic metastasis compared to tumors in other locations remains unexplored in this study, highlighting the need for further research.

TURBT is a crucial urological procedure and the standard treatment for NMIBC patients. Despite continuous improvements in instrument since its first report in 1910^20^, the fundamental concept remains consistent: completely remove visible bladder tumors and provide specimens with an adequate muscular layer for accurate diagnosis^3^. Previous studies have emphasized tumor residue as the primary factor for TURBT recurrence^21,22^, with ~17–67% of TA patients and 20–71% of T1 patients exhibiting residual tumors during repeat transurethral resection^22^. Moreover, a standardized TURBT surgical approach can significantly reduce the risk of recurrence^23^. However, tumors located in the bladder neck and anterior wall present challenges for transurethral resection, often leading to incomplete removal^10,24,25^. This may result in limited visibility during TURBT surgery for tumors on the anterior wall and bladder dome, potentially increasing the risk of residual tumors and operational difficulties. Operational challenges could also lead to tumor compression, raising the likelihood of tumor cells release and intravesical implantation. Hence, some propose cystectomy for challenging T1 stage NMIBC locations^26^. This study suggests that compared to standard TURBT treatment, partial cystectomy significantly enhances survival for dome tumors but not for anterior wall tumors. Recommendations for managing NMIBC patients with tumors in the anterior wall and dome include: (1) considering laparoscopic partial cystectomy for bladder dome tumors. (2) Improving TURBT instruments for enhanced flexibility, thorough tumor resection, and improved prognosis. (3) Exploring lateral or prone position surgery for anterior wall tumors with cautious preparation before clinical implementation.

Although this study, which possesses the largest sample size to date, delves into the impact of tumor location on NMIBC patient prognosis after TURBT, it does recognize its limitations. The scarcity of cases with partial cystectomy may compromise reliability and representativeness. The study is susceptible to selection and immortal-time bias. In the Chinese NMIBC cohort, the diversity of centers and follow-up methods may introduce bias, and the absence of precise surgery-to-diagnosis time in both cohorts raises the possibility of immortal-time bias. Furthermore, neither the Chinese NMIBC cohort nor the SEER database provides specific information regarding endoscopic instruments, visual enhancement technologies, surgeons’ skill levels, or postoperative adjuvant treatments. However, these factors could potentially influence the effectiveness of TURBT procedures and the detection of bladder tumors.

## Conclusion

Tumor location significantly influences TURBT outcomes in NMIBC patients, with anterior wall and bladder dome tumors having worse post-TURBT prognosis (earlier recurrence, shortened OS, and DSS); partial cystectomy improves outcomes for bladder dome tumors compared to TURBT. This study provides guidance for personalized treatment and prognosis management for NMIBC patients with tumors in different locations.

## Ethical approval

The study included 118 477 patients: 5569 from the Chinese NMIBC cohort (1996–2019) and 112 908 (TURBT: 112 666 cases; partial cystectomy: 242 cases) from the SEER database (2000–2020). The study received approval from the Ethics Review Committee of Tongji Hospital, Tongji Medical College, Huazhong University of Science and Technology (TJ-IRB20230888).

## Consent

The requirement for informed consent was also waived because the retrospective data analysis posed no additional harm to the patients.

## Sources of funding

This research was supported by the National Natural Science Foundation of China (No. 82303623), the National Sponsored Postdoctoral Research Program of China (No. GZB20230243), the China Postdoctoral Science Foundation (No. 2023M731199), the Postdoctoral Innovation Research Position of Hubei Province, China (Postdoctoral No. 331048), and the Tongji Hospital of Tongji Medical College, Huazhong University of Science and Technology, China (2022YBJ018, 2021RCYJ005). The study’s funder was not involved in the study design, data collection, data analysis, data interpretation, and decision to write or publish the manuscript.

## Author contribution

K.C., Z.L., J.H., and L.L.: conceptualization; L.L., K.C., Z.L., J.H., K.L., S.-g.W., Chinese Bladder Cancer Consortium, J.W., Z.Y., Y.X., Z.J., Z.C., H.H., H.C., T.C., J.B., Z.W., Y.W., P.Z., Q.W., Z.C., L.L., J.H., Y.H., Z.L., Y.L., Y.D., Y.K., Y.X., J.H., J.Z., and H.L.: data curation; L.L., K.L., S.-g.W., and J.W.: formal analysis; K.C. and L.L.: funding acquisition; L.L., K.L., S.-g.W., and J.W.: investigation; K.C., L.L., J.H., Y.H., and J.W.: methodology; Chinese Bladder Cancer Consortium, Y.X., Z.J., Z.C., H.H., H.C., T.C., J.B., Z.W., Y.W., P.Z., Q.W., Z.C., and L.L.: project administration; Chinese Bladder Cancer Consortium, J.H., K.L., S.-g.W., and Z.L.: resources; software: not applicable; K.C., Z.L., and J.H.: supervision; K.C., Z.L., J.H., S.-g.W., L.L., and J.W.: validation; L.L. and J.W.: visualization; L.L.: writing – original draft; K.C., L.L., Z.L., J.H., K.L., S.-g.W., J.W., Z.Y., Y.X., Z.J., Z.C., H.H., H.C., T.C., J.B., Z.W., Y.W., P.Z., Q.W., Z.C., L.L., J.H., Y.H., Z.L., Y.L., Y.D., Y.K., Y.X., J.H., J.Z., and H.L.: writing – review and editing. The ultimate version received approval for submission from all authors. The authors extend their gratitude to Professor Tao Jing (School of Public Health, Tongji Medical College, Huazhong University of Science and Technology, Wuhan, China) for providing statistical assistance.

## Conflicts of interest disclosure

The authors have no potential conflicts of interest to disclose.

## Research registration unique identifying number (UIN)

1. Name of the registry: ClinicalTrials.gov [the Protocol Registration and Results System (PRS)].
2. Unique identifying number or registration ID: NCT06245759.
3. Hyperlink to your specific registration: https://classic.clinicaltrials.gov/ct2/show/NCT06245759.

## Guarantor

Ke Chen, Zheng Liu, Jian Huang, and Lilong Liu had full access to all of the data in the study and take responsibility for the integrity of the data and the accuracy of the data analysis.

## Data availability statement

All detailed data are available in manuscript. The summary statistical data from the Chinese NMIBC cohort can be provided by the corresponding author upon any request. Data from the SEER cohort can be obtained following the SEER official guidelines.

## Provenance and peer review

Not commissioned, externally peer-reviewed.

## Supplementary Material
