# Supplementary material for: The prognostic impact of tumor location in nonmuscle-invasive bladder cancer patients undergoing transurethral resection: insights from a cohort study utilizing Chinese multicenter and SEER registries
Source: Int J Surg. 2024 May 24;110(9):5641–51. doi: 10.1097/JS9.0000000000001675 (PMC11392199; doi:10.1097/JS9.0000000000001675)

***Supplementary appendix***

**The Prognostic Impact of Tumor Location in Non-Muscle-Invasive Bladder Cancer Patients Undergoing Transurethral Resection: Insights from a Cohort Study Utilizing Chinese Multicenter and SEER Registries**

| **Table S1** | Comparison of baseline characteristics among patients with tumors in different locations in the Chinese NMIBC cohort | Page 2 |
| --- | --- | --- |
| **Table S2** | Comparison of baseline characteristics among patients with tumors in different locations in the SEER cohort | Page 4 |
| **Table S3** | Univariate and multivariate COX analysis of the overall survival of NMIBC patients in the Chinese NMIBC cohort | Page 7 |
| **Table S4** | Univariate and multivariate COX analysis of the disease-specific survival of NMIBC patients in the SEER database | Page 9 |
| **Table S5** | Partial cystectomy vs TURBT of NMIBC located in dome (SEER database) | Page 11 |
| **Table S6** | Partial cystectomy vs TURBT of NMIBC located in anterior wall (SEER database) | Page 13 |
| **Figure S1** | Flow diagram of Chinese NMIBC cohort and SEER cohort patient inclusion and exclusion process | Page 15 |
| **Figure S2** | The flow diagram of patient inclusion and exclusion processes for TURBT and partial cystectomy in NMIBC patients within the SEER cohort | Page 16 |
| **Figure S3** | Comparing the prognosis of patients with tumors in different locations in the Chinese NMIBC cohort, stratified based on age | Page 17 |
| **Figure S4** | Comparing the prognosis of patients with tumors in different locations in the SEER cohort, stratified based on age | Page 18 |
| **Figure S5** | Comparing the prognosis of patients with tumors in different locations in the Chinese NMIBC cohort, stratified based on gender | Page 19 |
| **Figure S6** | Comparing the prognosis of patients with tumors in different locations in the SEER cohort, stratified based on gender | Page 20 |
| **Figure S7** | Comparing the prognosis of patients with tumors in different locations in the Chinese NMIBC cohort, stratified based on T value | Page 21 |
| **Figure S8** | Comparing the prognosis of patients with tumors in different locations in the SEER cohort, stratified based on T value | Page 22 |
| **Figure S9** | Comparing the prognosis of patients with tumors in different locations in the Chinese NMIBC cohort, stratified based on Grade | Page 23 |
| **Figure S10** | Comparing the prognosis of patients with tumors in different locations in the SEER cohort, stratified based on Grade | Page 24 |
| **Figure S11** | Comparing the prognosis of patients with tumors in different locations in the Chinese NMIBC cohort, stratified based on number of tumors | Page 25 |
| **Figure S12** | Comparing the prognosis of patients with tumors in different locations in the SEER cohort, stratified based on Grade | Page 26 |
| **Figure S13** | Comparing the prognosis of patients with tumors in different locations in the SEER cohort, stratified based on tumor size | Page 27 |
| **Figure S14** | Univariate COX analysis of the recurrence-free survival of NMIBC patients in the Chinese NMIBC cohort | Page 28 |
| **Figure S15** | Univariate COX analysis of the overall survival of NMIBC patients in the SEER cohort | Page 29 |

**Table S1 Comparison of baseline characteristics among patients with tumors in different locations in the Chinese NMIBC cohort.**

| **Characteristics** | **Overall** | **Lateral wall** | **Anterior wall** | **Bladder neck** | **Dome** | **Overlapping lesion** | **Posterior wall** | **Trigone** | **Urachus** | **Ureteric orifice** | **p** |
| --- | --- | --- | --- | --- | --- | --- | --- | --- | --- | --- | --- |
| n (%) | 5569 (100) | 1644 (29.52) | 193 (3.47) | 229 (4.11) | 257 (4.61) | 1794 (32.21) | 465 (8.35) | 333 (5.98) | 7 (0.13) | 647 (11.62) |  |
| **Age (%)** |  |  |  |  |  |  |  |  |  |  |  |
| 18-64 | 2812 (50.49) | 862 (52.43) | 87 (45.08) | 112 (48.91) | 113 (43.97) | 903 (50.33) | 237 (50.97) | 173 (51.95) | 3 (42.86) | 322 (49.77) | 0.2658 |
| 65+ | 2757 (49.51) | 782 (47.57) | 106 (54.92) | 117 (51.09) | 144 (56.03) | 891 (49.67) | 228 (49.03) | 160 (48.05) | 4 (57.14) | 325 (50.23) | |
| **Gender (%)** | |  |  |  |  |  |  |  |  |  |  |
| Male | 4447 (79.85) | 1338 (81.39) | 157 (81.35) | 191 (83.41) | 209 (81.32) | 1440 (80.27) | 341 (73.33) | 264 (79.28) | 5 (71.43) | 502 (77.59) | 0.0114 |
| Female | 1122 (20.15) | 306 (18.61) | 36 (18.65) | 38 (16.59) | 48 (18.68) | 354 (19.73) | 124 (26.67) | 69 (20.72) | 2 (28.57) | 145 (22.41) | |
| **Tumors (%)** | |  |  |  |  |  |  |  |  |  |  |
| Single | 2045 (36.72) | 912 (55.47) | 89 (46.11) | 125 (54.59) | 121 (47.08) | 0 (0.00) | 262 (56.34) | 171 (51.35) | 3 (42.86) | 362 (55.95) | <0.0001 |
| Multi | 2744 (49.27) | 409 (24.88) | 61 (31.61) | 60 (26.20) | 78 (30.35) | 1794 (100.00) | 92 (19.78) | 93 (27.93) | 1 (14.29) | 156 (24.11) | |
| Unknown | 780 (14.01) | 323 (19.65) | 43 (22.28) | 44 (19.21) | 58 (22.57) | 0 (0.00) | 111 (23.87) | 69 (20.72) | 3 (42.86) | 129 (19.94) | |
| **Diagnosis (%)** | |  |  |  |  |  |  |  |  |  |  |
| 1996-2012 | 2542 (45.65) | 760 (46.23) | 89 (46.11) | 88 (38.43) | 121 (47.08) | 843 (46.99) | 187 (40.22) | 166 (49.85) | 4 (57.14) | 284 (43.89) | 0.0478 |
| 2013+ | 3027 (54.35) | 884 (53.77) | 104 (53.89) | 141 (61.57) | 136 (52.92) | 951 (53.01) | 278 (59.78) | 167 (50.15) | 3 (42.86) | 363 (56.11) | |
| **Tvalue (%)** |  |  |  |  |  |  |  |  |  |  |  |
| TA | 1769 (31.77) | 651 (39.60) | 47 (24.35) | 60 (26.20) | 50 (19.46) | 481 (26.81) | 165 (35.48) | 95 (28.53) | 3 (42.86) | 217 (33.54) | <0.0001 |
| TIS | 291 (5.23) | 105 (6.39) | 5 (2.59) | 14 (6.11) | 22 (8.56) | 71 (3.96) | 28 (6.02) | 13 (3.90) | 0 (0.00) | 33 (5.10) |  |
| T1 | 2056 (36.92) | 542 (32.97) | 99 (51.30) | 88 (38.43) | 106 (41.25) | 682 (38.02) | 171 (36.77) | 144 (43.24) | 4 (57.14) | 220 (34.00) | |
| Unknown | 1453 (26.09) | 346 (21.05) | 42 (21.76) | 67 (29.26) | 79 (30.74) | 560 (31.22) | 101 (21.72) | 81 (24.32) | 0 (0.00) | 177 (27.36) | |
| **Grade WHO 2004 (%)** | | | | | | | | | | | |
| Low | 1426 (25.61) | 427 (25.97) | 36 (18.65) | 56 (24.45) | 50 (19.46) | 488 (27.20) | 118 (25.38) | 69 (20.72) | 1 (14.29) | 181 (27.98) | <0.0001 |
| High | 1691 (30.36) | 466 (28.35) | 69 (35.75) | 64 (27.95) | 93 (36.19) | 583 (32.50) | 121 (26.02) | 87 (26.13) | 2 (28.57) | 206 (31.84) | |
| PUNLMP | 601 (10.79) | 192 (11.68) | 23 (11.92) | 23 (10.04) | 28 (10.89) | 127 (7.08) | 78 (16.77) | 40 (12.01) | 0 (0.00) | 90 (13.91) |  |
| Unknown | 1851 (33.24) | 559 (34.00) | 65 (33.68) | 86 (37.55) | 86 (33.46) | 596 (33.22) | 148 (31.83) | 137 (41.14) | 4 (57.14) | 170 (26.28) | |
| **Histology (%)** | |  |  |  |  |  |  |  |  |  |  |
| TCC | 5107 (91.70) | 1504 (91.48) | 175 (90.67) | 214 (93.45) | 235 (91.44) | 1653 (92.14) | 434 (93.33) | 304 (91.29) | 3 (42.86) | 585 (90.42) | <0.0001 |
| SCC | 109 (1.96) | 25 (1.52) | 5 (2.59) | 5 (2.18) | 7 (2.72) | 37 (2.06) | 11 (2.37) | 7 (2.10) | 0 (0.00) | 12 (1.85) |  |
| ACC | 82 (1.47) | 26 (1.58) | 5 (2.59) | 3 (1.31) | 3 (1.17) | 22 (1.23) | 6 (1.29) | 6 (1.80) | 3 (42.86) | 8 (1.24) |  |
| Others | 271 (4.87) | 89 (5.41) | 8 (4.15) | 7 (3.06) | 12 (4.67) | 82 (4.57) | 14 (3.01) | 16 (4.80) | 1 (14.29) | 42 (6.49) |  |

Abbreviations: TCC: Urothelial carcinoma; ACC: Adenocarcinoma; SCC: Squamous carcinoma. The p values represent differences among patients with tumors in different locations, and a p value < 0.05 indicates significant differences.

**Table S2 Comparison of baseline characteristics among patients with tumors in different locations in the SEER cohort.**

| **Characteristics** | **Overall** | **Lateral wall** | **Anterior wall** | **Bladder neck** | **Dome** | **Overlapping lesion** | **Posterior wall** | **Trigone** | **Urachus** | **Ureteric orifice** | **p** |
| --- | --- | --- | --- | --- | --- | --- | --- | --- | --- | --- | --- |
| n (%) | 112666 (100) | 42966 (38.14) | 3484 (3.09) | 5441 (4.83) | 5679 (5.04) | 15965 (14.17) | 18432 (16.36) | 12402 (11.01) | 28 (0.02) | 8269 (7.34) |  |
| **Age (%)** |  |  |  |  |  |  |  |  |  |  |  |
| 18-64 | 31688 (28.13) | 13086 (30.46) | 716 (20.55) | 1425 (26.19) | 1097 (19.32) | 4058 (25.42) | 4750 (25.77) | 3664 (29.54) | 11 (39.29) | 2881 (34.84) | <0.0001 |
| 65+ | 80978 (71.87) | 29880 (69.54) | 2768 (79.45) | 4016 (73.81) | 4582 (80.68) | 11907 (74.58) | 13682 (74.23) | 8738 (70.46) | 17 (60.71) | 5388 (65.16) | |
| **Gender (%)** | |  |  |  |  |  |  |  |  |  |  |
| Male | 87248 (77.44) | 33045 (76.91) | 2902 (83.30) | 4560 (83.81) | 4674 (82.30) | 12425 (77.83) | 14292 (77.54) | 9341 (75.32) | 20 (71.43) | 5989 (72.43) | <0.0001 |
| Female | 25418 (22.56) | 9921 (23.09) | 582 (16.70) | 881 (16.19) | 1005 (17.70) | 3540 (22.17) | 4140 (22.46) | 3061 (24.68) | 8 (28.57) | 2280 (27.57) | |
| **Race (%)** |  |  |  |  |  |  |  |  |  |  |  |
| White | 101786 (90.34) | 38933 (90.61) | 3053 (87.63) | 4812 (88.44) | 5003 (88.10) | 14515 (90.92) | 16635 (90.25) | 11239 (90.62) | 25 (89.29) | 7571 (91.56) | <0.0001 |
| Black | 5012 (4.45) | 1765 (4.11) | 233 (6.69) | 301 (5.53) | 344 (6.06) | 767 (4.80) | 747 (4.05) | 562 (4.53) | 2 (7.14) | 291 (3.52) |  |
| Other | 5224 (4.64) | 1997 (4.65) | 184 (5.28) | 297 (5.46) | 293 (5.16) | 619 (3.88) | 951 (5.16) | 524 (4.23) | 1 (3.57) | 358 (4.33) |  |
| Unknown | 644 (0.57) | 271 (0.63) | 14 (0.40) | 31 (0.57) | 39 (0.69) | 64 (0.40) | 99 (0.54) | 77 (0.62) | 0 (0.00) | 49 (0.59) |  |
| **Rural.Urban (%)** | |  |  |  |  |  |  |  |  |  |  |
| Metropolitan | 98517 (87.49) | 37316 (86.92) | 3071 (88.15) | 4740 (87.18) | 5047 (88.92) | 13896 (87.09) | 16226 (88.07) | 10945 (88.28) | 25 (89.29) | 7251 (87.72) | <0.0001 |
| Nonmetropolitan | 14086 (12.51) | 5617 (13.08) | 413 (11.85) | 697 (12.82) | 629 (11.08) | 2060 (12.91) | 2199 (11.93) | 1453 (11.72) | 3 (10.71) | 1015 (12.28) | |
| **Income (%)** | |  |  |  |  |  |  |  |  |  |  |
| <35000 | 848 (0.75) | 373 (0.87) | 14 (0.40) | 42 (0.77) | 40 (0.70) | 124 (0.78) | 129 (0.70) | 79 (0.64) | 0 (0.00) | 47 (0.57) | <0.0001 |
| 35000-74999 | 60965 (54.11) | 23517 (54.74) | 1943 (55.77) | 3001 (55.16) | 3016 (53.11) | 8805 (55.15) | 9550 (51.81) | 6555 (52.85) | 23 (82.14) | 4555 (55.09) | |
| 75000+ | 50850 (45.13) | 19073 (44.39) | 1527 (43.83) | 2398 (44.07) | 2623 (46.19) | 7036 (44.07) | 8753 (47.49) | 5768 (46.51) | 5 (17.86) | 3667 (44.35) | |
| **Number.of.tumors (%)** | | |  |  |  |  |  |  |  |  |  |
| Single | 67989 (60.35) | 26844 (62.48) | 2048 (58.78) | 2892 (53.15) | 3141 (55.32) | 9683 (60.66) | 10929 (59.30) | 7467 (60.21) | 18 (64.29) | 4967 (60.07) | <0.0001 |
| Multi | 44671 (39.65) | 16122 (37.52) | 1436 (41.22) | 2549 (46.85) | 2537 (44.68) | 6281 (39.34) | 7501 (40.70) | 4934 (39.79) | 10 (35.71) | 3301 (39.93) | |
| **Radiation (%)** | |  |  |  |  |  |  |  |  |  |  |
| Yes | 667 (0.59) | 187 (0.44) | 34 (0.98) | 40 (0.74) | 53 (0.93) | 167 (1.05) | 94 (0.51) | 72 (0.58) | 0 (0.00) | 20 (0.24) | <0.0001 |
| No/Unknown | 111999 (99.41) | 42779 (99.56) | 3450 (99.02) | 5401 (99.26) | 5626 (99.07) | 15798 (98.95) | 18338 (99.49) | 12330 (99.42) | 28 (100.00) | 8249 (99.76) | |
| **Chemotherapy (%)** | |  |  |  |  |  |  |  |  |  |  |
| Yes | 18065 (16.03) | 7215 (16.79) | 610 (17.51) | 730 (13.42) | 948 (16.69) | 2620 (16.41) | 2920 (15.84) | 1896 (15.29) | 4 (14.29) | 1122 (13.57) | <0.0001 |
| No/Unknown | 94601 (83.97) | 35751 (83.21) | 2874 (82.49) | 4711 (86.58) | 4731 (83.31) | 13345 (83.59) | 15512 (84.16) | 10506 (84.71) | 24 (85.71) | 7147 (86.43) | |
| **Year.of.diagnosis (%)** | |  |  |  |  |  |  |  |  |  |  |
| 2000-2009 | 52236 (46.36) | 19202 (44.69) | 1485 (42.62) | 2602 (47.82) | 2566 (45.18) | 7683 (48.12) | 8614 (46.73) | 5621 (45.32) | 15 (53.57) | 4448 (53.79) | <0.0001 |
| 2010-2020 | 60430 (53.64) | 23764 (55.31) | 1999 (57.38) | 2839 (52.18) | 3113 (54.82) | 8282 (51.88) | 9818 (53.27) | 6781 (54.68) | 13 (46.43) | 3821 (46.21) | |
| **Tvalue (%)** |  |  |  |  |  |  |  |  |  |  |  |
| TA | 74568 (66.19) | 29906 (69.60) | 1963 (56.34) | 3348 (61.53) | 3038 (53.50) | 8961 (56.13) | 12111 (65.71) | 8746 (70.52) | 14 (50.00) | 6481 (78.38) | <0.0001 |
| TIS | 6108 (5.42) | 2107 (4.90) | 185 (5.31) | 337 (6.19) | 382 (6.73) | 868 (5.44) | 1234 (6.69) | 630 (5.08) | 4 (14.29) | 361 (4.37) |  |
| T1 | 31990 (28.39) | 10953 (25.49) | 1336 (38.35) | 1756 (32.27) | 2259 (39.78) | 6136 (38.43) | 5087 (27.60) | 3026 (24.40) | 10 (35.71) | 1427 (17.26) | |
| **Grade (%)** |  |  |  |  |  |  |  |  |  |  |  |
| Grade I+II | 42020 (37.30) | 17213 (40.06) | 942 (27.04) | 1879 (34.53) | 1475 (25.97) | 5073 (31.78) | 6591 (35.76) | 5094 (41.07) | 6 (21.43) | 3747 (45.31) | <0.0001 |
| Grade III+IV | 40376 (35.84) | 14184 (33.01) | 1773 (50.89) | 2131 (39.17) | 2919 (51.40) | 7082 (44.36) | 6823 (37.02) | 3794 (30.59) | 1 (3.57) | 1669 (20.18) | |
| Unknown | 30270 (26.87) | 11569 (26.93) | 769 (22.07) | 1431 (26.30) | 1285 (22.63) | 3810 (23.86) | 5018 (27.22) | 3514 (28.33) | 21 (75.00) | 2853 (34.50) | |
| **Histologic.Type (%)** | |  |  |  |  |  |  |  |  |  |  |
| TCC | 111077 (98.59) | 42489 (98.89) | 3404 (97.70) | 5348 (98.29) | 5545 (97.64) | 15720 (98.47) | 18117 (98.29) | 12239 (98.69) | 16 (57.14) | 8199 (99.15) | <0.0001 |
| SCC | 769 (0.68) | 250 (0.58) | 41 (1.18) | 36 (0.66) | 48 (0.85) | 134 (0.84) | 137 (0.74) | 77 (0.62) | 0 (0.00) | 46 (0.56) |  |
| ACC | 257 (0.23) | 54 (0.13) | 12 (0.34) | 31 (0.57) | 32 (0.56) | 33 (0.21) | 51 (0.28) | 28 (0.23) | 6 (21.43) | 10 (0.12) |  |
| Others | 563 (0.50) | 173 (0.40) | 27 (0.77) | 26 (0.48) | 54 (0.95) | 78 (0.49) | 127 (0.69) | 58 (0.47) | 6 (21.43) | 14 (0.17) |  |
| **Tumor Size (%)** | |  |  |  |  |  |  |  |  |  |  |
| <=20 mm | 20090 (17.83) | 8191 (19.06) | 619 (17.77) | 860 (15.81) | 1074 (18.91) | 1470 (9.21) | 3784 (20.53) | 2247 (18.12) | 4 (14.29) | 1841 (22.26) | <0.0001 |
| 21+ mm | 31422 (27.89) | 12733 (29.64) | 1046 (30.02) | 1060 (19.48) | 1445 (25.44) | 5252 (32.90) | 4801 (26.05) | 3212 (25.90) | 7 (25.00) | 1866 (22.57) | |
| Unknown | 61154 (54.28) | 22042 (51.30) | 1819 (52.21) | 3521 (64.71) | 3160 (55.64) | 9243 (57.90) | 9847 (53.42) | 6943 (55.98) | 17 (60.71) | 4562 (55.17) | |

Abbreviations: SEER: Surveillance, Epidemiology, and End Results. TCC: Urothelial carcinoma; ACC: Adenocarcinoma; SCC: Squamous carcinoma. The p values represent differences among patients with tumors in different locations, and a p value < 0.05 indicates significant differences.

**Table S3 Univariate and multivariate COX analysis of the overall survival of NMIBC patients in the Chinese NMIBC cohort.**

| **Characteristics** | **Univariate COX analysis** | | | **Multivariable COX analysis** | | |
| --- | --- | --- | --- | --- | --- | --- |
|  | **HR** | **95% CI** | **P** | **HR** | **95% CI** | **P** |
| **Age** |  |  |  |  |  |  |
| 18-64 | Reference |  |  | Reference |  |  |
| 65+ | 2.72 | 2.05-3.6 | <0.0001 | 2.33 | 1.75 - 3.09 | <0.0001 |
| **Gender** |  |  |  |  |  |  |
| Male | Reference |  |  | Reference |  |  |
| Female | 0.95 | 0.68-1.32 | 0.742 |  |  |  |
| **Diagnosis** |  |  |  |  |  |  |
| 1996-2012 | Reference |  |  | Reference |  |  |
| 2013+ | 0.42 | 0.32-0.56 | <0.0001 | 0.47 | 0.35 - 0.63 | <0.0001 |
| **Primary.Site** |  |  |  |  |  |  |
| Lateral wall | Reference |  |  | Reference |  |  |
| Anterior wall | 5.9 | 3.33-10.45 | <0.0001 | 4.35 | 2.44 - 7.77 | <0.0001 |
| Bladder neck | 1.91 | 1-3.64 | 0.049 | 1.69 | 0.88 - 3.25 | 0.113 |
| Dome | 9.56 | 5.92-15.45 | <0.0001 | 7.91 | 4.84 - 12.94 | <0.0001 |
| Overlapping lesion | 2.77 | 1.94-3.95 | <0.0001 | 1.7 | 1 - 2.9 | 0.0514 |
| Posterior wall | 0.8 | 0.43-1.5 | 0.492 | 0.99 | 0.53 - 1.85 | 0.9716 |
| Trigone | 2.59 | 1.47-4.59 | 0.001 | 2.06 | 1.16 - 3.67 | 0.0138 |
| Urachus | 3.69 | 0.51-26.74 | 0.197 | 2.92 | 0.38 - 22.4 | 0.3037 |
| Ureteric orifice | 1.46 | 0.91-2.34 | 0.114 | 1.49 | 0.93 - 2.39 | 0.0998 |
| **Number.of.tumors** |  |  |  |  |  |  |
| Single | Reference |  |  | Reference |  |  |
| Multi | 3.68 | 2.58-5.25 | <0.0001 | 3.53 | 2.08 - 5.98 | <0.0001 |
| Unknown | 5.24 | 3.69-7.43 | <0.0001 | 5.41 | 3.78 - 7.75 | <0.0001 |
| **Tvalue** |  |  |  |  |  |  |
| TA | Reference |  |  | Reference |  |  |
| TIS | 1.01 | 0.64-1.61 | 0.951 | 0.87 | 0.54 - 1.41 | 0.5796 |
| T1 | 1.46 | 1.09-1.94 | 0.01 | 1.37 | 1.02 - 1.84 | 0.037 |
| Unknown | 0 | 0-Inf | 0.991 | 0 | 0 - Inf | 0.9918 |
| **Grade WHO 2004** |  |  |  |  |  |  |
| Low | Reference |  |  | Reference |  |  |
| High | 2 | 1.33-3.03 | 0.001 | 1.67 | 1.1 - 2.55 | 0.0172 |
| PUNLMP | 1.31 | 0.85-2 | 0.222 | 1.29 | 0.84 - 2 | 0.2449 |
| Unknown | 2.44 | 1.65-3.61 | <0.0001 | 2.27 | 1.52 - 3.4 | 0.0001 |
| **Histology** |  |  |  |  |  |  |
| TCC | Reference |  |  | Reference |  |  |
| SCC | 0.87 | 0.28-2.72 | 0.809 | 0.62 | 0.19 - 1.98 | 0.4192 |
| ACC | 2.25 | 1.11-4.57 | 0.025 | 2.58 | 1.24 - 5.36 | 0.0112 |
| Others | 2.02 | 1.26-3.23 | 0.004 | 2.01 | 1.23 - 3.27 | 0.0051 |

Abbreviations: HR: hazard ratios; CI: confidence interval; TCC: Urothelial carcinoma; ACC: Adenocarcinoma; SCC: Squamous carcinoma. The p values represent differences among patients with tumors in different locations, and a p value < 0.05 indicates significant differences.

**Table S4 Univariate and multivariate COX analysis of the disease-specific survival of NMIBC patients in the SEER database.**

| **Characteristics** | **Univariate COX analysis** | | | **Multivariable COX analysis** | | |
| --- | --- | --- | --- | --- | --- | --- |
|  | **HR** | **95% CI** | **P** | **HR** | **95% CI** | **P** |
| **Age** |  |  |  |  |  |  |
| 18-64 | Reference |  |  | Reference |  |  |
| 65+ | 3.35 | 3.19-3.52 | <0.0001 | 2.9 | 2.76 - 3.06 | <0.0001 |
| **Gender** |  |  |  |  |  |  |
| Male | Reference |  |  | Reference |  |  |
| Female | 0.96 | 0.92-1 | 0.071 |  |  |  |
| **Race** |  |  |  |  |  |  |
| White | Reference |  |  | Reference |  |  |
| Black | 1.34 | 1.24-1.45 | <0.0001 | 1.33 | 1.23 - 1.44 | <0.0001 |
| Other | 0.91 | 0.84-1 | 0.048 | 0.92 | 0.84 - 1.01 | 0.0751 |
| Unknown | 0.22 | 0.13-0.38 | <0.0001 | 0.34 | 0.2 - 0.59 | 0.0001 |
| **Rural.Urban** |  |  |  |  |  |  |
| Metropolitan | Reference |  |  | Reference |  |  |
| Nonmetropolitan | 1.12 | 1.06-1.18 | <0.0001 | 1.15 | 1.09 - 1.21 | <0.0001 |
| **Income** |  |  |  |  |  |  |
| <35000 | Reference |  |  |  |  |  |
| 35000-74999 | 0.93 | 0.76-1.14 | 0.49 |  |  |  |
| 75000+ | 0.84 | 0.69-1.03 | 0.091 |  |  |  |
| **Number.of.tumors** |  |  |  |  |  |  |
| Single | Reference |  |  | Reference |  |  |
| Multi | 1.68 | 1.62-1.74 | <0.0001 | 1.48 | 1.43 - 1.53 | <0.0001 |
| **Primary.Site** |  |  |  |  |  |  |
| Lateral wall | Reference |  |  | Reference |  |  |
| Anterior wall | 1.52 | 1.38-1.68 | <0.0001 | 1.13 | 1.02 - 1.24 | 0.0183 |
| Bladder neck | 1.68 | 1.56-1.81 | <0.0001 | 1.39 | 1.28 - 1.5 | <0.0001 |
| Dome | 1.62 | 1.5-1.75 | <0.0001 | 1.14 | 1.06 - 1.24 | 0.0006 |
| Overlapping lesion | 1.79 | 1.7-1.88 | <0.0001 | 1.43 | 1.36 - 1.51 | <0.0001 |
| Posterior wall | 1.16 | 1.1-1.22 | <0.0001 | 1.05 | 0.99 - 1.11 | 0.094 |
| Trigone | 1.19 | 1.11-1.26 | <0.0001 | 1.18 | 1.11 - 1.26 | <0.0001 |
| Urachus | 0.65 | 0.16-2.6 | 0.543 | 0.49 | 0.12 - 1.96 | 0.3112 |
| Ureteric orifice | 0.88 | 0.81-0.95 | 0.001 | 1.05 | 0.97 - 1.13 | 0.2502 |
| **Radiation** |  |  |  |  |  |  |
| Yes | Reference |  |  | Reference |  |  |
| No/Unknown | 0.14 | 0.13-0.16 | <0.0001 | 0.32 | 0.29 - 0.36 | <0.0001 |
| **Chemotherapy** |  |  |  |  |  |  |
| Yes | Reference |  |  |  |  |  |
| No/Unknown | 1.03 | 0.98-1.09 | 0.205 |  |  |  |
| **Year.of.diagnosis** |  |  |  |  |  |  |
| 2000-2009 | Reference |  |  |  |  |  |
| 2010-2020 | 1.03 | 0.99-1.07 | 0.111 |  |  |  |
| **Tvalue** |  |  |  |  |  |  |
| TA | Reference |  |  | Reference |  |  |
| TIS | 1.8 | 1.67-1.94 | <0.0001 | 1.52 | 1.41 - 1.64 | <0.0001 |
| T1 | 2.93 | 2.83-3.04 | <0.0001 | 2.12 | 2.03 - 2.21 | <0.0001 |
| **Grade** |  |  |  |  |  |  |
| Grade I+II | Reference |  |  | Reference |  |  |
| Grade III+IV | 2.58 | 2.47-2.69 | <0.0001 | 1.71 | 1.64 - 1.79 | <0.0001 |
| Unknown | 1.09 | 1.04-1.15 | 0.001 | 1.08 | 1.02 - 1.13 | 0.0044 |
| **Histologic.Type** |  |  |  |  |  |  |
| TCC | Reference |  |  | Reference |  |  |
| SCC | 2.22 | 1.92-2.56 | <0.0001 | 2.09 | 1.8 - 2.41 | <0.0001 |
| ACC | 3.13 | 2.47-3.95 | <0.0001 | 2 | 1.58 - 2.53 | <0.0001 |
| Others | 2.73 | 2.31-3.21 | <0.0001 | 1.68 | 1.41 - 1.99 | <0.0001 |
| **Tumor Size (mm)** |  |  |  |  |  |  |
| <=20 | Reference |  |  | Reference |  |  |
| Size21+ | 1.48 | 1.39-1.58 | <0.0001 | 1.21 | 1.13 - 1.29 | <0.0001 |
| Unknown | 1.54 | 1.45-1.63 | <0.0001 | 1.31 | 1.24 - 1.39 | <0.0001 |

Abbreviations: HR: hazard ratios; CI: confidence interval; TCC: Urothelial carcinoma; ACC: Adenocarcinoma; SCC: Squamous carcinoma. The p values represent differences among patients with tumors in different locations, and a p value < 0.05 indicates significant differences.

**Table S5 Partial cystectomy vs TURBT of NMIBC located in dome (SEER database).**

| **Characteristics** | **Before PSM** | | | | **After PSM** | | | |
| --- | --- | --- | --- | --- | --- | --- | --- | --- |
|  | **Overall** | **TURBT** | **Partial cystectomy** | **p** | **Overall** | **TURBT** | **Partial cystectomy** | **p** |
| n | 5867 | 5678 | 189 |  | 378 | 189 | 189 |  |
| **Age (%)** |  |  |  |  |  |  |  |  |
| 18-39 | 50 (0.85) | 46 (0.81) | 4 (2.12) | 0.0001 | 6 (1.59) | 2 (1.06) | 4 (2.12) | 0.1301 |
| 40-64 | 1105 (18.83) | 1050 (18.49) | 55 (29.10) |  | 95 (25.13) | 40 (21.16) | 55 (29.10) |  |
| 65+ | 4712 (80.31) | 4582 (80.70) | 130 (68.78) | | 277 (73.28) | 147 (77.78) | 130 (68.78) | |
| **Gender (%)** | |  |  |  |  |  |  |  |
| Male | 4810 (81.98) | 4673 (82.30) | 137 (72.49) | 0.0008 | 287 (75.93) | 150 (79.37) | 137 (72.49) | 0.1488 |
| Female | 1057 (18.02) | 1005 (17.70) | 52 (27.51) |  | 91 (24.07) | 39 (20.63) | 52 (27.51) |  |
| **Race (%)** |  |  |  |  |  |  |  |  |
| White | 5160 (87.95) | 5002 (88.09) | 158 (83.60) | 0.1474 | 322 (85.19) | 164 (86.77) | 158 (83.60) | 0.629 |
| Black | 363 (6.19) | 344 (6.06) | 19 (10.05) |  | 31 (8.20) | 12 (6.35) | 19 (10.05) |  |
| Other | 304 (5.18) | 293 (5.16) | 11 (5.82) |  | 23 (6.08) | 12 (6.35) | 11 (5.82) |  |
| Unknown | 40 (0.68) | 39 (0.69) | 1 (0.53) |  | 2 (0.53) | 1 (0.53) | 1 (0.53) |  |
| **Rural.Urban (%)** | |  |  |  |  |  |  |  |
| Metropolitan | 5209 (88.78) | 5047 (88.89) | 162 (85.71) | 0.3657 | 335 (88.62) | 173 (91.53) | 162 (85.71) | 0.1053 |
| Nonmetropolitan | 655 (11.16) | 628 (11.06) | 27 (14.29) |  | 43 (11.38) | 16 (8.47) | 27 (14.29) |  |
| Unknown | 3 (0.05) | 3 (0.05) | 0 (0.00) |  |  |  |  |  |
| **Income (%)** | |  |  |  |  |  |  |  |
| <35000 | 40 (0.68) | 40 (0.70) | 0 (0.00) | 0.3111 |  |  |  | 0.2563 |
| 35000-74999 | 3123 (53.23) | 3015 (53.10) | 108 (57.14) | | 204 (53.97) | 96 (50.79) | 108 (57.14) |  |
| 75000+ | 2704 (46.09) | 2623 (46.20) | 81 (42.86) |  | 174 (46.03) | 93 (49.21) | 81 (42.86) |  |
| **Number.of.tumors (%)** | | |  |  |  |  |  |  |
| Single | 3253 (55.45) | 3141 (55.32) | 112 (59.26) | 0.555 | 209 (55.29) | 97 (51.32) | 112 (59.26) | 0.1475 |
| Multi | 2613 (44.54) | 2536 (44.66) | 77 (40.74) |  | 169 (44.71) | 92 (48.68) | 77 (40.74) |  |
| Unknown | 1 (0.02) | 1 (0.02) | 0 (0.00) |  |  |  |  |  |
| **Radiation (%)** | |  |  |  |  |  |  |  |
| Yes | 56 (0.95) | 53 (0.93) | 3 (1.59) | 0.5966 | 6 (1.59) | 3 (1.59) | 3 (1.59) | 1 |
| No/Unknown | 5811 (99.05) | 5625 (99.07) | 186 (98.41) | | 372 (98.41) | 186 (98.41) | 186 (98.41) | |
| **Chemotherapy (%)** | |  |  |  |  |  |  |  |
| Yes | 963 (16.41) | 948 (16.70) | 15 (7.94) | 0.0019 | 29 (7.67) | 14 (7.41) | 15 (7.94) | 1 |
| No/Unknown | 4904 (83.59) | 4730 (83.30) | 174 (92.06) | | 349 (92.33) | 175 (92.59) | 174 (92.06) | |
| **Year.of.diagnosis (%)** | |  |  |  |  |  |  |  |
| 2000-2004 | 1383 (23.57) | 1330 (23.42) | 53 (28.04) | 0.0792 | 103 (27.25) | 50 (26.46) | 53 (28.04) | 0.7533 |
| 2005-2009 | 1283 (21.87) | 1235 (21.75) | 48 (25.40) |  | 102 (26.98) | 54 (28.57) | 48 (25.40) |  |
| 2010-2014 | 1436 (24.48) | 1390 (24.48) | 46 (24.34) |  | 85 (22.49) | 39 (20.63) | 46 (24.34) |  |
| 2015-2020 | 1765 (30.08) | 1723 (30.35) | 42 (22.22) |  | 88 (23.28) | 46 (24.34) | 42 (22.22) |  |
| **Tvalue (%)** |  |  |  |  |  |  |  |  |
| TA | 3090 (52.67) | 3038 (53.50) | 52 (27.51) | <0.0001 | 105 (27.78) | 53 (28.04) | 52 (27.51) | 0.9819 |
| TIS | 401 (6.83) | 382 (6.73) | 19 (10.05) |  | 37 (9.79) | 18 (9.52) | 19 (10.05) |  |
| T1 | 2376 (40.50) | 2258 (39.77) | 118 (62.43) | | 236 (62.43) | 118 (62.43) | 118 (62.43) | |
| **Grade (%)** |  |  |  |  |  |  |  |  |
| Grade I | 536 (9.14) | 523 (9.21) | 13 (6.88) | 0.1249 | 29 (7.67) | 16 (8.47) | 13 (6.88) | 0.9133 |
| Grade II | 1298 (22.12) | 1262 (22.23) | 36 (19.05) |  | 76 (20.11) | 40 (21.16) | 36 (19.05) |  |
| Grade III | 1006 (17.15) | 961 (16.92) | 45 (23.81) |  | 88 (23.28) | 43 (22.75) | 45 (23.81) |  |
| Grade IV | 1434 (24.44) | 1387 (24.43) | 47 (24.87) |  | 95 (25.13) | 48 (25.40) | 47 (24.87) |  |
| Unknown | 1593 (27.15) | 1545 (27.21) | 48 (25.40) |  | 90 (23.81) | 42 (22.22) | 48 (25.40) |  |
| **Histologic.Type (%)** | |  |  |  |  |  |  |  |
| TCC | 5690 (96.98) | 5544 (97.64) | 146 (77.25) | <0.0001 | 287 (75.93) | 141 (74.60) | 146 (77.25) | 0.7802 |
| SCC | 51 (0.87) | 48 (0.85) | 3 (1.59) |  | 6 (1.59) | 3 (1.59) | 3 (1.59) |  |
| ACC | 62 (1.06) | 32 (0.56) | 30 (15.87) |  | 60 (15.87) | 30 (15.87) | 30 (15.87) |  |
| Others | 64 (1.09) | 54 (0.95) | 10 (5.29) |  | 25 (6.61) | 15 (7.94) | 10 (5.29) |  |
| **Tumorsize (%)** | |  |  |  |  |  |  |  |
| <=10 | 402 (6.85) | 385 (6.78) | 17 (8.99) | 0.5955 | 37 (9.79) | 20 (10.58) | 17 (8.99) | 0.7639 |
| 10-20 | 710 (12.10) | 689 (12.13) | 21 (11.11) |  | 43 (11.38) | 22 (11.64) | 21 (11.11) |  |
| 21-50 | 1283 (21.87) | 1237 (21.79) | 46 (24.34) |  | 96 (25.40) | 50 (26.46) | 46 (24.34) |  |
| 50+ | 213 (3.63) | 208 (3.66) | 5 (2.65) |  | 7 (1.85) | 2 (1.06) | 5 (2.65) |  |
| Unknown | 3259 (55.55) | 3159 (55.64) | 100 (52.91) | | 195 (51.59) | 95 (50.26) | 100 (52.91) | |

Abbreviations: SEER: Surveillance, Epidemiology, and End Results. PSM: propensity score matching; TCC: Urothelial carcinoma; ACC: Adenocarcinoma; SCC: Squamous carcinoma. The p values represent differences among patients with tumors in different locations, and a p value < 0.05 indicates significant differences.

**Table S6 Partial cystectomy vs TURBT of NMIBC located in anterior wall (SEER database).**

| **Characteristics** | **Before PSM** | | | | **After PSM** | | | |
| --- | --- | --- | --- | --- | --- | --- | --- | --- |
|  | **Overall** | **TURBT** | **Partial cystectomy** | **p** | **Overall** | **TURBT** | **Partial cystectomy** | **p** |
| n | 3537 | 3484 | 53 |  | 106 | 53 | 53 |  |
| **Age (%)** |  |  |  |  |  |  |  |  |
| 18-39 | 33 (0.93) | 32 (0.92) | 1 (1.89) | 0.6476 | 1 (0.94) | 0 (0.00) | 1 (1.89) | 0.5004 |
| 40-64 | 696 (19.68) | 684 (19.63) | 12 (22.64) |  | 27 (25.47) | 15 (28.30) | 12 (22.64) |  |
| 65+ | 2808 (79.39) | 2768 (79.45) | 40 (75.47) |  | 78 (73.58) | 38 (71.70) | 40 (75.47) |  |
| **Gender (%)** | |  |  |  |  |  |  |  |
| Male | 2949 (83.38) | 2902 (83.30) | 47 (88.68) | 0.3903 | 99 (93.40) | 52 (98.11) | 47 (88.68) | 0.1177 |
| Female | 588 (16.62) | 582 (16.70) | 6 (11.32) |  | 7 (6.60) | 1 (1.89) | 6 (11.32) |  |
| **Race (%)** |  |  |  |  |  |  |  |  |
| White | 3102 (87.70) | 3053 (87.63) | 49 (92.45) | 0.7424 | 96 (90.57) | 47 (88.68) | 49 (92.45) | 0.7018 |
| Black | 235 (6.64) | 233 (6.69) | 2 (3.77) |  | 6 (5.66) | 4 (7.55) | 2 (3.77) |  |
| Other | 186 (5.26) | 184 (5.28) | 2 (3.77) |  | 4 (3.77) | 2 (3.77) | 2 (3.77) |  |
| Unknown | 14 (0.40) | 14 (0.40) | 0 (0.00) |  |  |  |  |  |
| **Rural.Urban (%)** | |  |  |  |  |  |  |  |
| Metropolitan | 3117 (88.13) | 3071 (88.15) | 46 (86.79) | 0.9296 | 93 (87.74) | 47 (88.68) | 46 (86.79) | 1 |
| Nonmetropolitan | 420 (11.87) | 413 (11.85) | 7 (13.21) |  | 13 (12.26) | 6 (11.32) | 7 (13.21) |  |
| **Income (%)** | |  |  |  |  |  |  |  |
| <35000 | 14 (0.40) | 14 (0.40) | 0 (0.00) | 0.8079 |  |  |  |  |
| 35000-74999 | 1971 (55.73) | 1943 (55.77) | 28 (52.83) |  | 51 (48.11) | 23 (43.40) | 28 (52.83) | 0.4368 |
| 75000+ | 1552 (43.88) | 1527 (43.83) | 25 (47.17) |  | 55 (51.89) | 30 (56.60) | 25 (47.17) |  |
| **Number.of.tumors (%)** | | |  |  |  |  |  |  |
| Single | 2075 (58.67) | 2048 (58.78) | 27 (50.94) | 0.3126 | 59 (55.66) | 32 (60.38) | 27 (50.94) | 0.4342 |
| Multi | 1462 (41.33) | 1436 (41.22) | 26 (49.06) |  | 47 (44.34) | 21 (39.62) | 26 (49.06) |  |
| **Radiation (%)** | |  |  |  |  |  |  |  |
| Yes | 35 (0.99) | 34 (0.98) | 1 (1.89) | 1 | 1 (0.94) | 0 (0.00) | 1 (1.89) | 1 |
| No/Unknown | 3502 (99.01) | 3450 (99.02) | 52 (98.11) |  | 105 (99.06) | 53 (100.00) | 52 (98.11) |  |
| **Chemotherapy (%)** | |  |  |  |  |  |  |  |
| Yes | 625 (17.67) | 610 (17.51) | 15 (28.30) | 0.0624 | 32 (30.19) | 17 (32.08) | 15 (28.30) | 0.8324 |
| No/Unknown | 2912 (82.33) | 2874 (82.49) | 38 (71.70) |  | 74 (69.81) | 36 (67.92) | 38 (71.70) |  |
| **Year.of.diagnosis (%)** | |  |  |  |  |  |  |  |
| 2000-2004 | 695 (19.65) | 679 (19.49) | 16 (30.19) | 0.2598 | 31 (29.25) | 15 (28.30) | 16 (30.19) | 0.9754 |
| 2005-2009 | 818 (23.13) | 806 (23.13) | 12 (22.64) |  | 24 (22.64) | 12 (22.64) | 12 (22.64) |  |
| 2010-2014 | 861 (24.34) | 850 (24.40) | 11 (20.75) |  | 21 (19.81) | 10 (18.87) | 11 (20.75) |  |
| 2015-2020 | 1163 (32.88) | 1149 (32.98) | 14 (26.42) |  | 30 (28.30) | 16 (30.19) | 14 (26.42) |  |
| **Tvalue (%)** |  |  |  |  |  |  |  |  |
| TA | 1976 (55.87) | 1963 (56.34) | 13 (24.53) | <0.0001 | 22 (20.75) | 9 (16.98) | 13 (24.53) | 0.6305 |
| TIS | 186 (5.26) | 185 (5.31) | 1 (1.89) |  | 2 (1.89) | 1 (1.89) | 1 (1.89) |  |
| T1 | 1375 (38.87) | 1336 (38.35) | 39 (73.58) |  | 82 (77.36) | 43 (81.13) | 39 (73.58) |  |
| **Grade (%)** |  |  |  |  |  |  |  |  |
| Grade I | 312 (8.82) | 310 (8.90) | 2 (3.77) | <0.0001 | 2 (1.89) | 0 (0.00) | 2 (3.77) | 0.5916 |
| Grade II | 806 (22.79) | 797 (22.88) | 9 (16.98) |  | 16 (15.09) | 7 (13.21) | 9 (16.98) |  |
| Grade III | 555 (15.69) | 539 (15.47) | 16 (30.19) |  | 33 (31.13) | 17 (32.08) | 16 (30.19) |  |
| Grade IV | 882 (24.94) | 859 (24.66) | 23 (43.40) |  | 47 (44.34) | 24 (45.28) | 23 (43.40) |  |
| Unknown | 982 (27.76) | 979 (28.10) | 3 (5.66) |  | 8 (7.55) | 5 (9.43) | 3 (5.66) |  |
| **Histologic.Type (%)** | |  |  |  |  |  |  |  |
| TCC | 3454 (97.65) | 3404 (97.70) | 50 (94.34) | 0.0205 | 102 (96.23) | 52 (98.11) | 50 (94.34) | 0.5034 |
| SCC | 41 (1.16) | 41 (1.18) | 0 (0.00) |  |  |  |  |  |
| ACC | 13 (0.37) | 12 (0.34) | 1 (1.89) |  | 1 (0.94) | 0 (0.00) | 1 (1.89) |  |
| Others | 29 (0.82) | 27 (0.77) | 2 (3.77) |  | 3 (2.83) | 1 (1.89) | 2 (3.77) |  |
| **Tumorsize (%)** | |  |  |  |  |  |  |  |
| <=10 | 215 (6.08) | 212 (6.08) | 3 (5.66) | 0.615 | 4 (3.77) | 1 (1.89) | 3 (5.66) | 0.6273 |
| 10-20 | 411 (11.62) | 407 (11.68) | 4 (7.55) |  | 7 (6.60) | 3 (5.66) | 4 (7.55) |  |
| 21-50 | 901 (25.47) | 883 (25.34) | 18 (33.96) |  | 36 (33.96) | 18 (33.96) | 18 (33.96) |  |
| 50+ | 166 (4.69) | 163 (4.68) | 3 (5.66) |  | 4 (3.77) | 1 (1.89) | 3 (5.66) |  |
| Unknown | 1844 (52.13) | 1819 (52.21) | 25 (47.17) |  | 55 (51.89) | 30 (56.60) | 25 (47.17) |  |

Abbreviations: SEER: Surveillance, Epidemiology, and End Results. PSM: propensity score matching; TCC: Urothelial carcinoma; ACC: Adenocarcinoma; SCC: Squamous carcinoma. The p values represent differences among patients with tumors in different locations, and a p value < 0.05 indicates significant differences.

**Figure S1 Flow diagram of Chinese NMIBC cohort and SEER cohort patient inclusion and exclusion process.**


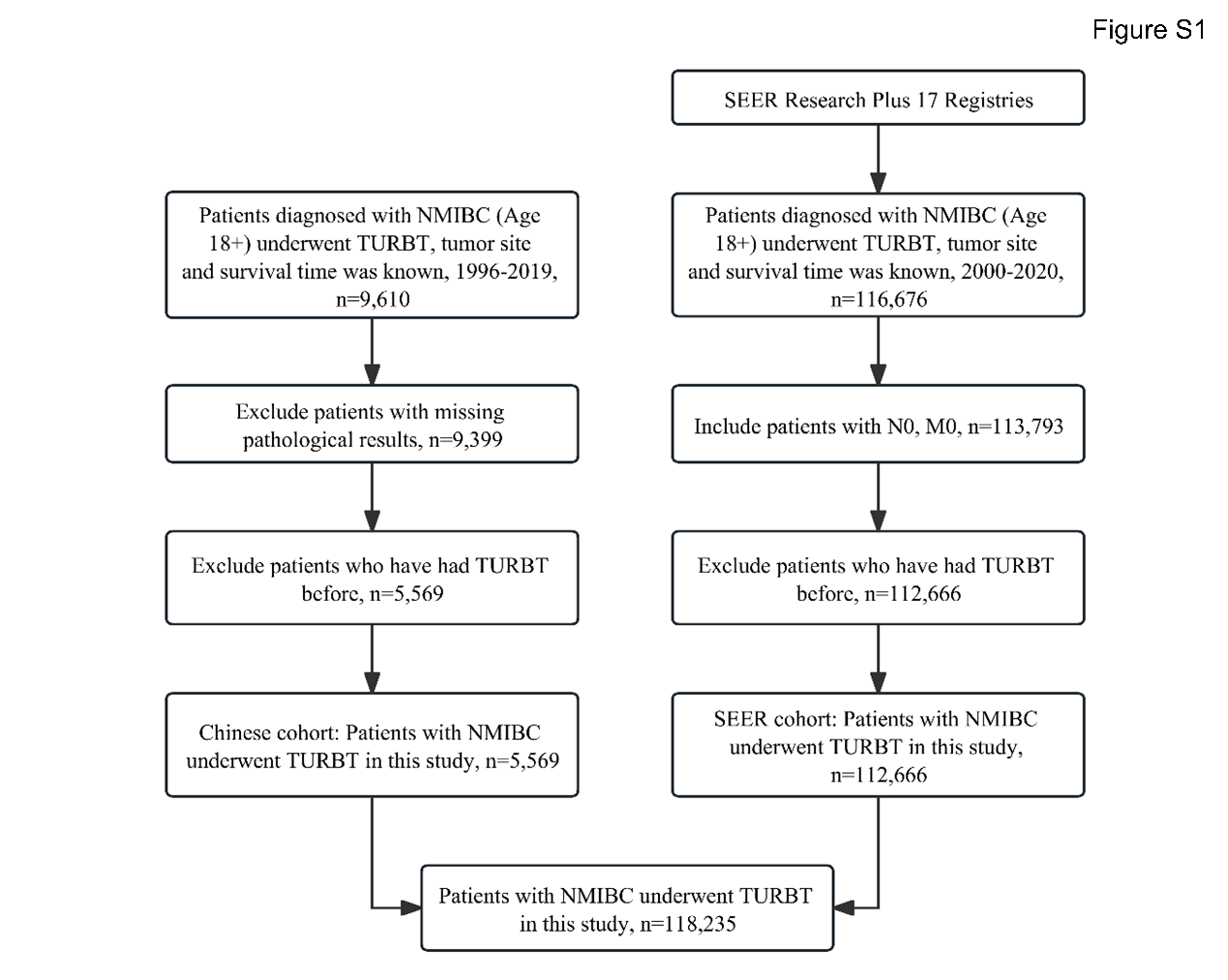
**Figure S2 The flow diagram of patient inclusion and exclusion processes for TURBT and partial cystectomy in NMIBC patients within the SEER cohort.**


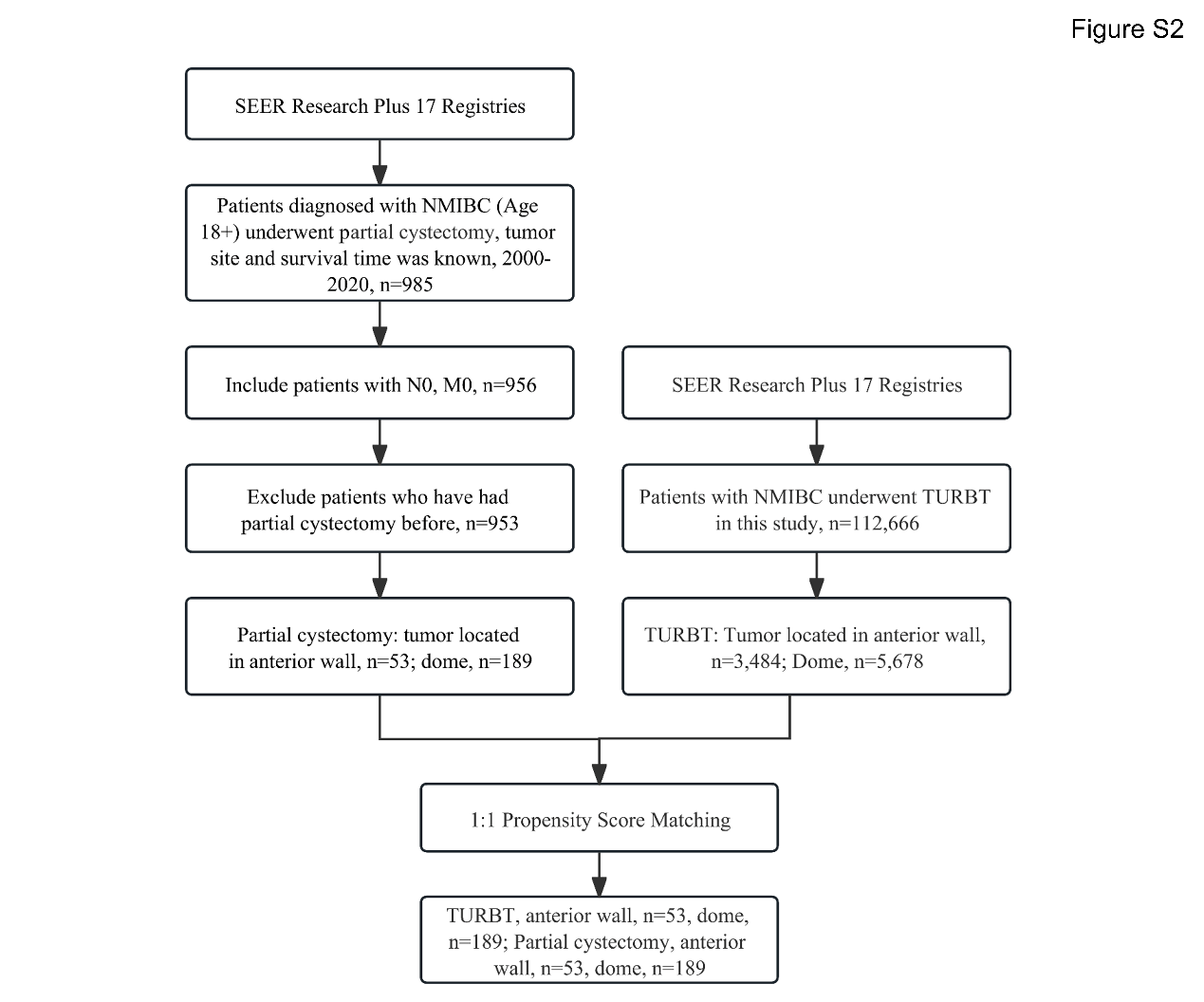


**Figure S3 Comparing the prognosis of patients with tumors in different locations in the Chinese NMIBC cohort, stratified based on age.** The overall survival curve and recurrence-free survival curve for patients aged 18-64 years (A-B) and 65+ years (C-D).


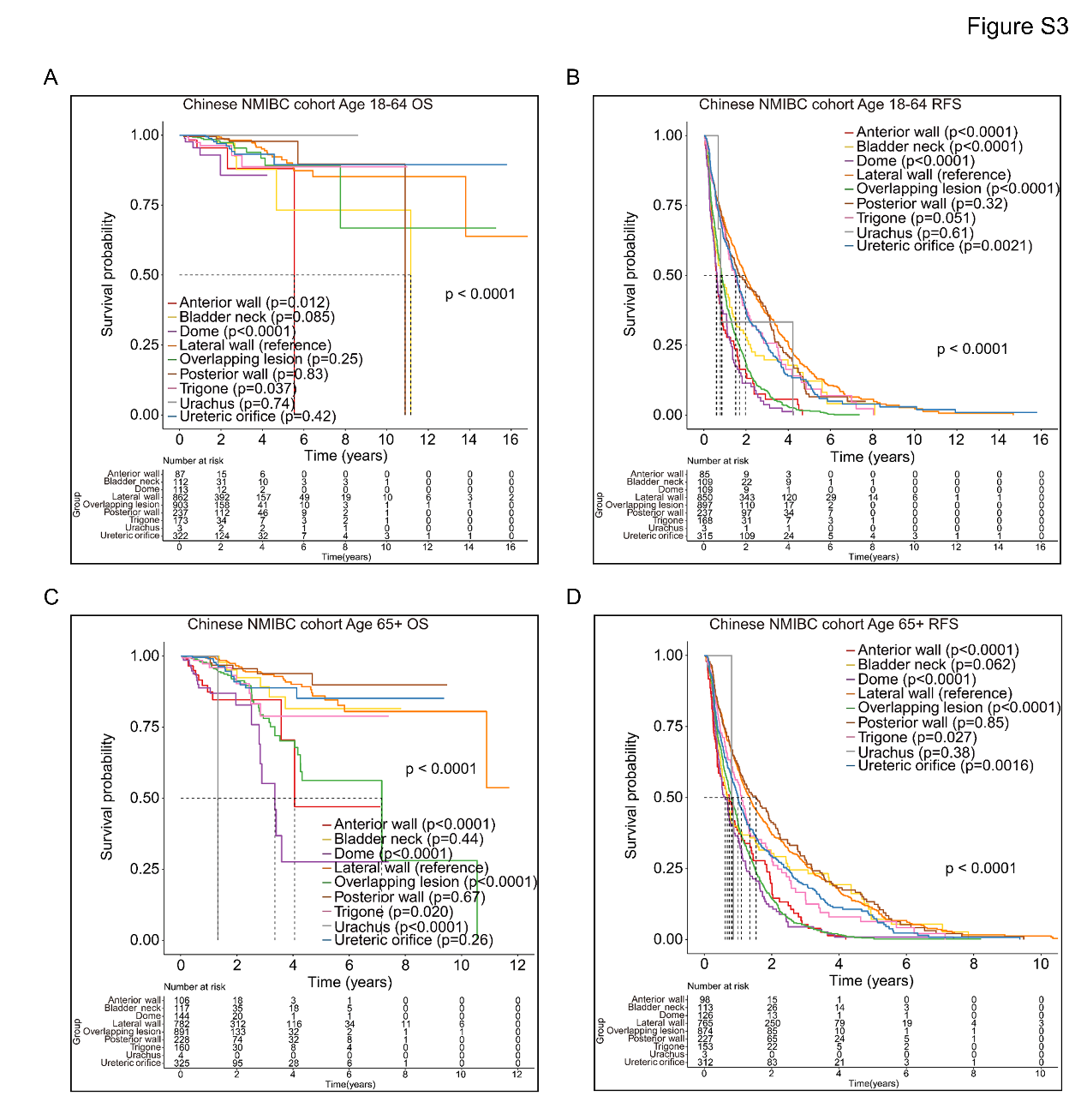


**Figure S4 Comparing the prognosis of patients with tumors in different locations in the SEER cohort, stratified based on age.** The overall survival curve and disease-specific survival curve for patients aged 18-64 years (A-B) and 65+ years (C-D).


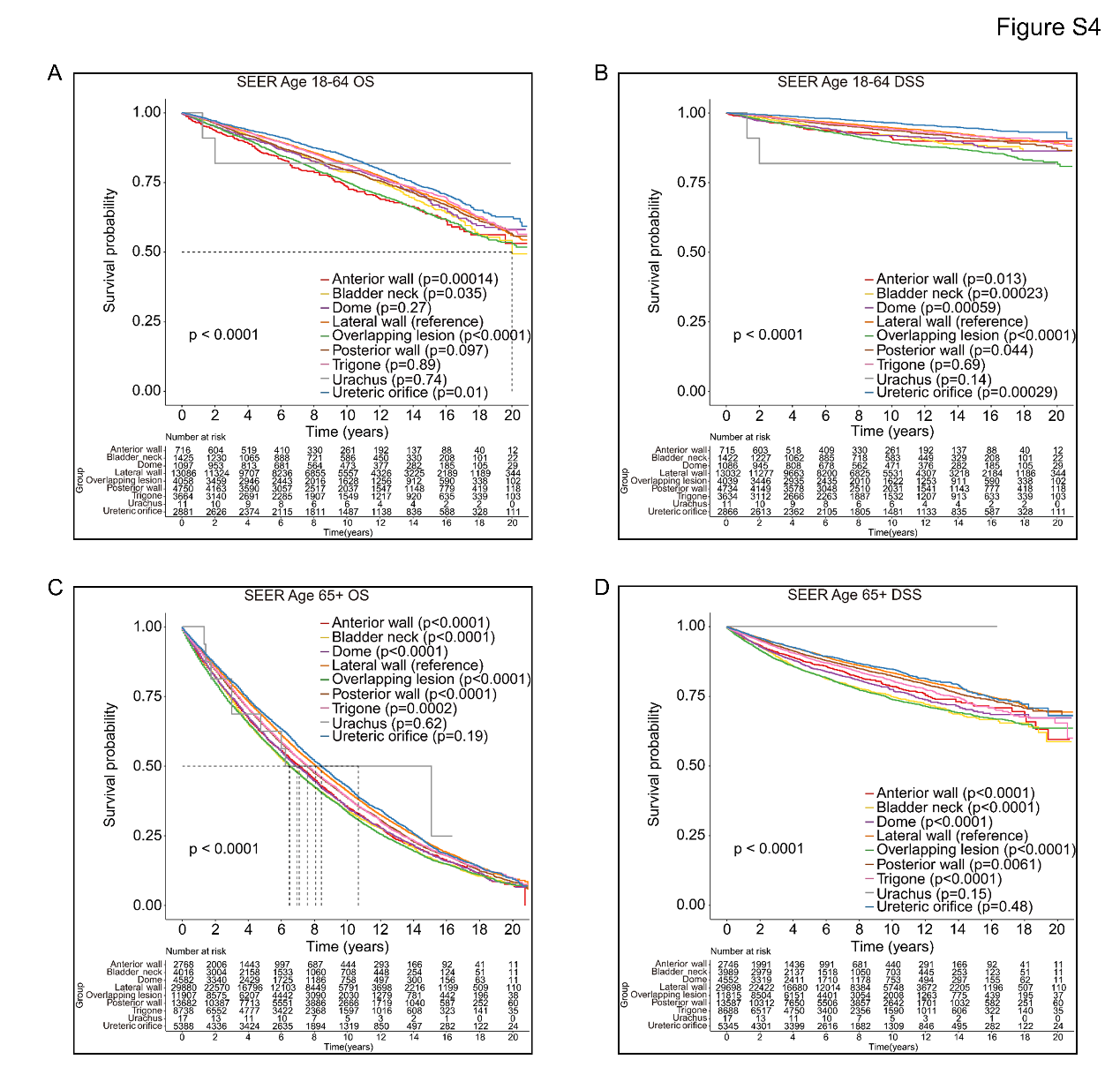


**Figure S5 Comparing the prognosis of patients with tumors in different locations in the Chinese NMIBC cohort, stratified based on gender.** The overall survival curve and recurrence-free survival curve for male patients (A-B) and female patients (C-D).


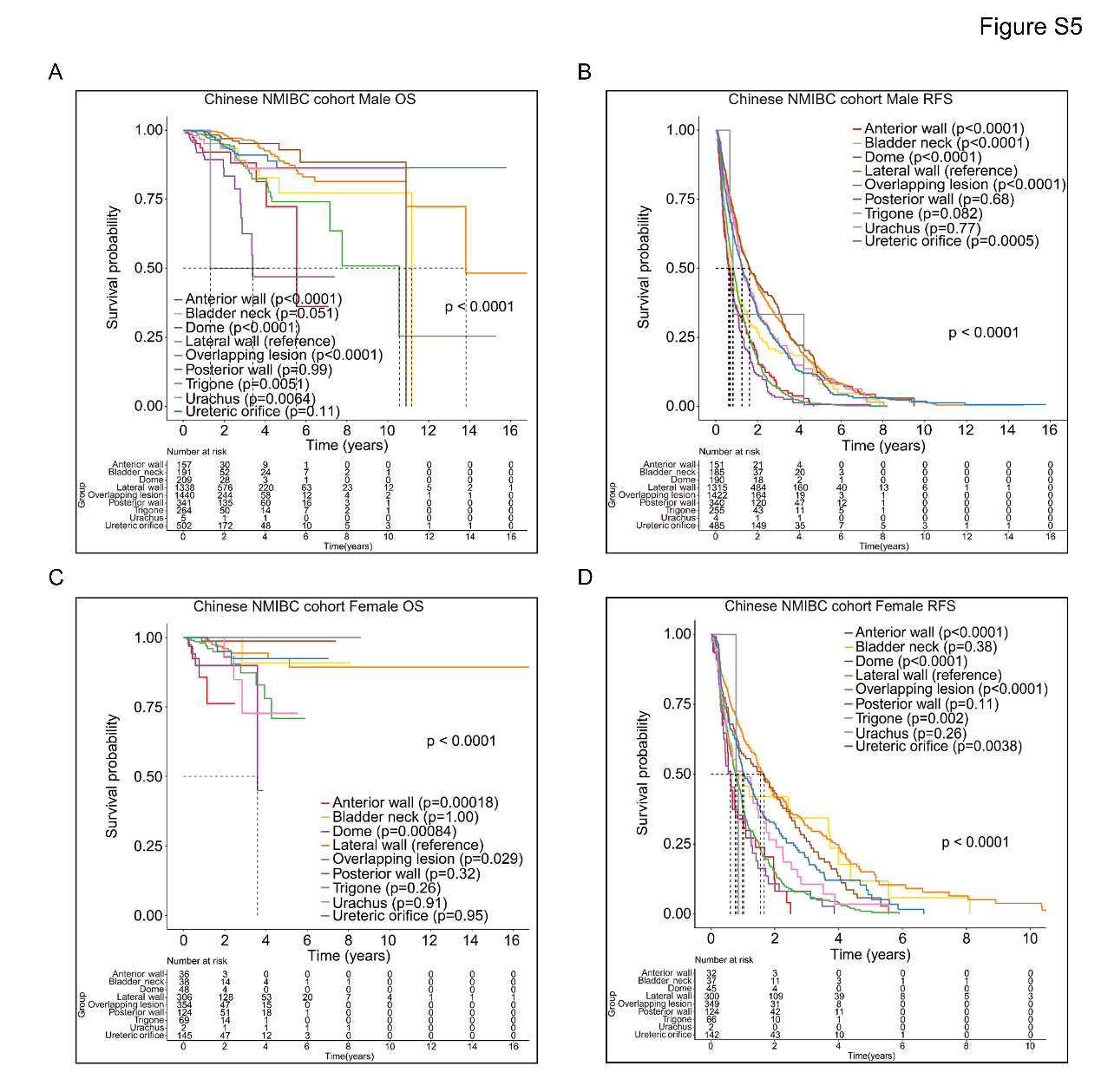


**Figure S6 Comparing the prognosis of patients with tumors in different locations in the SEER cohort, stratified based on gender.** The overall survival curve and disease-specific survival curve for male patients (A-B) and female patients (C-D).


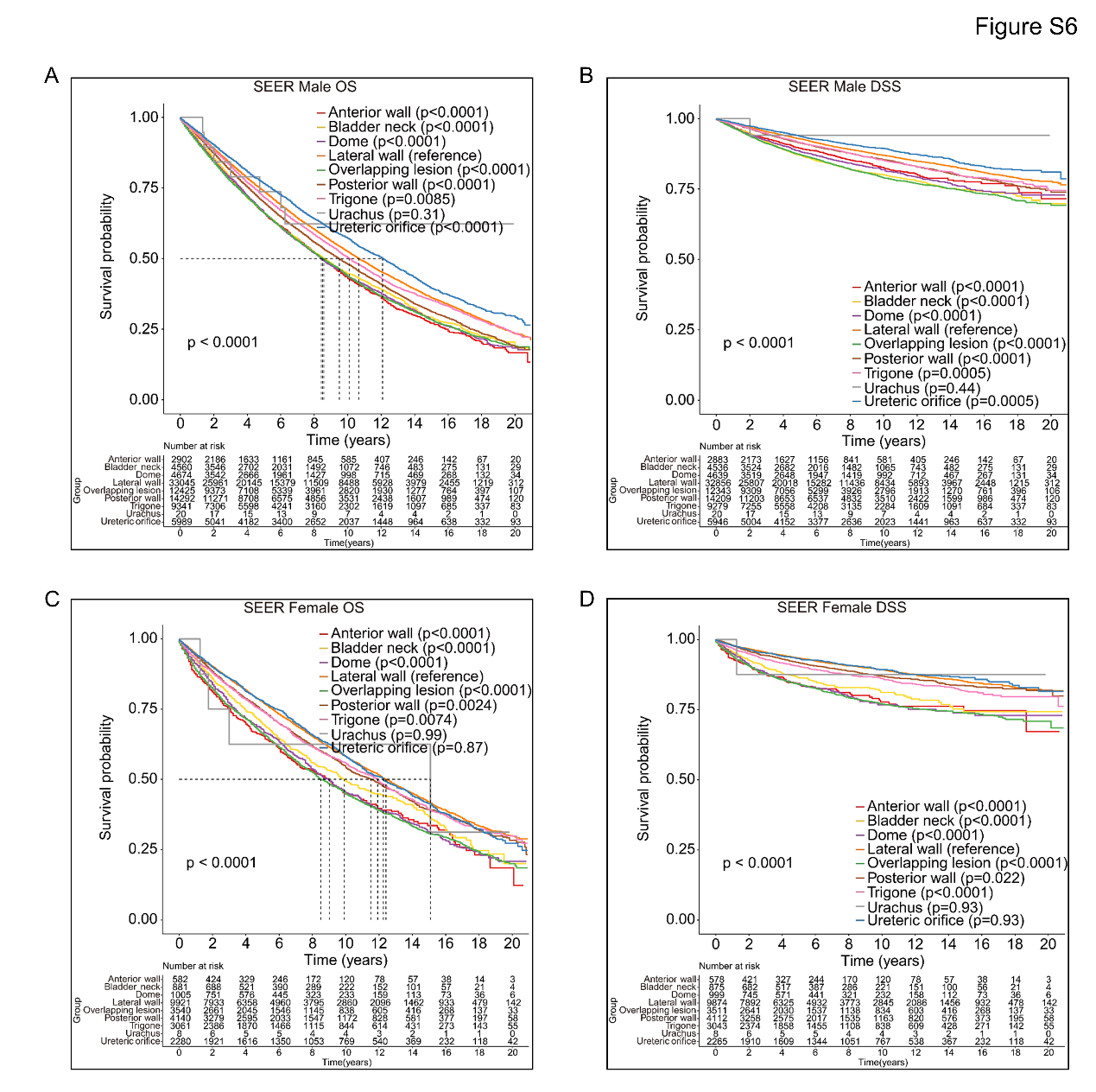


**Figure S7 Comparing the prognosis of patients with tumors in different locations in the Chinese NMIBC cohort, stratified based on T value.** The overall survival curve and recurrence-free survival curve for TA (A-B), TIS (C-D), and T1 (E-F) patients.


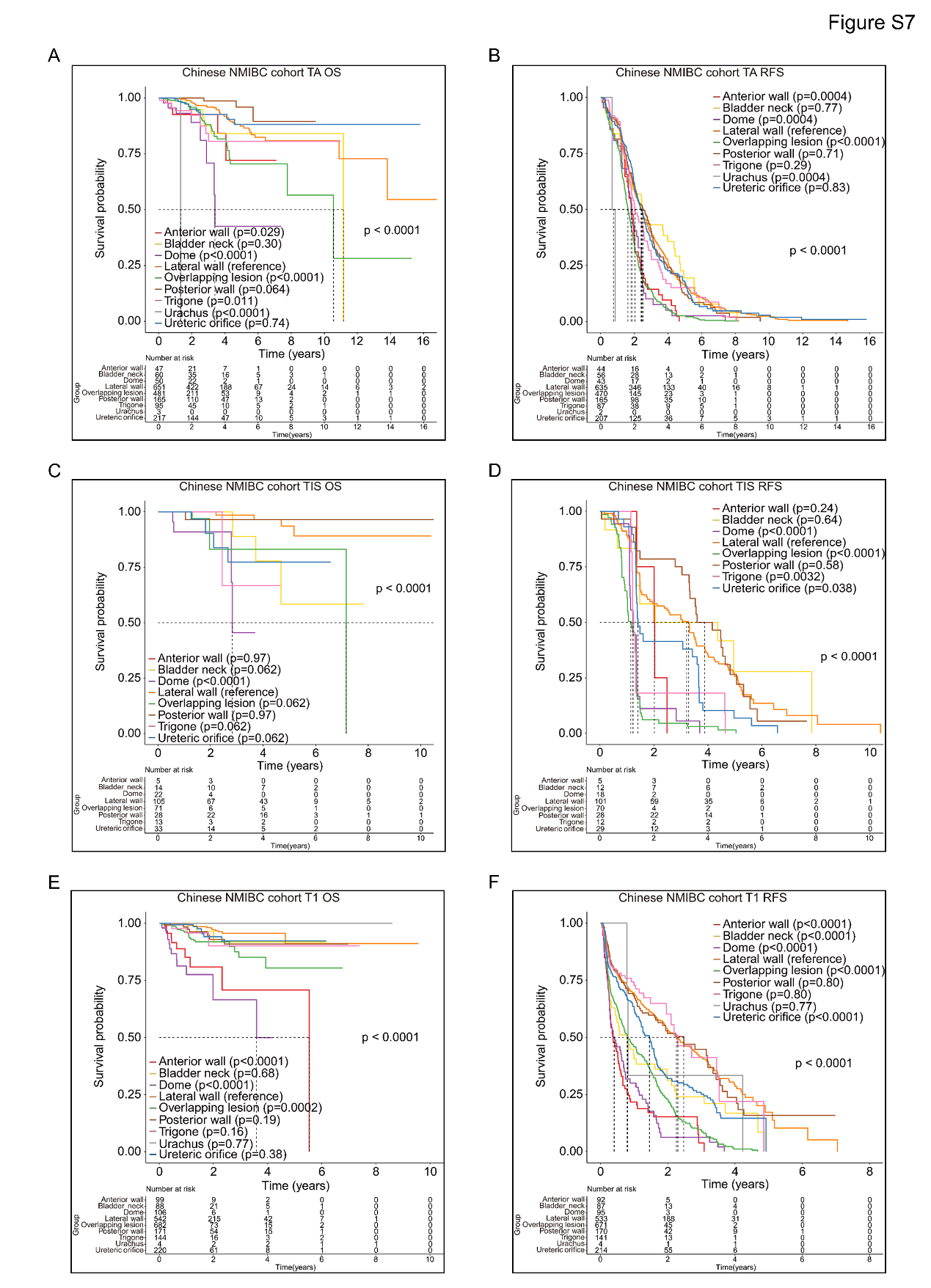


**Figure S8 Comparing the prognosis of patients with tumors in different locations in the SEER cohort, stratified based on T value.** The overall survival curve and disease-specific survival curve for TA (A-B), TIS (C-D), and T1 (E-F) patients.


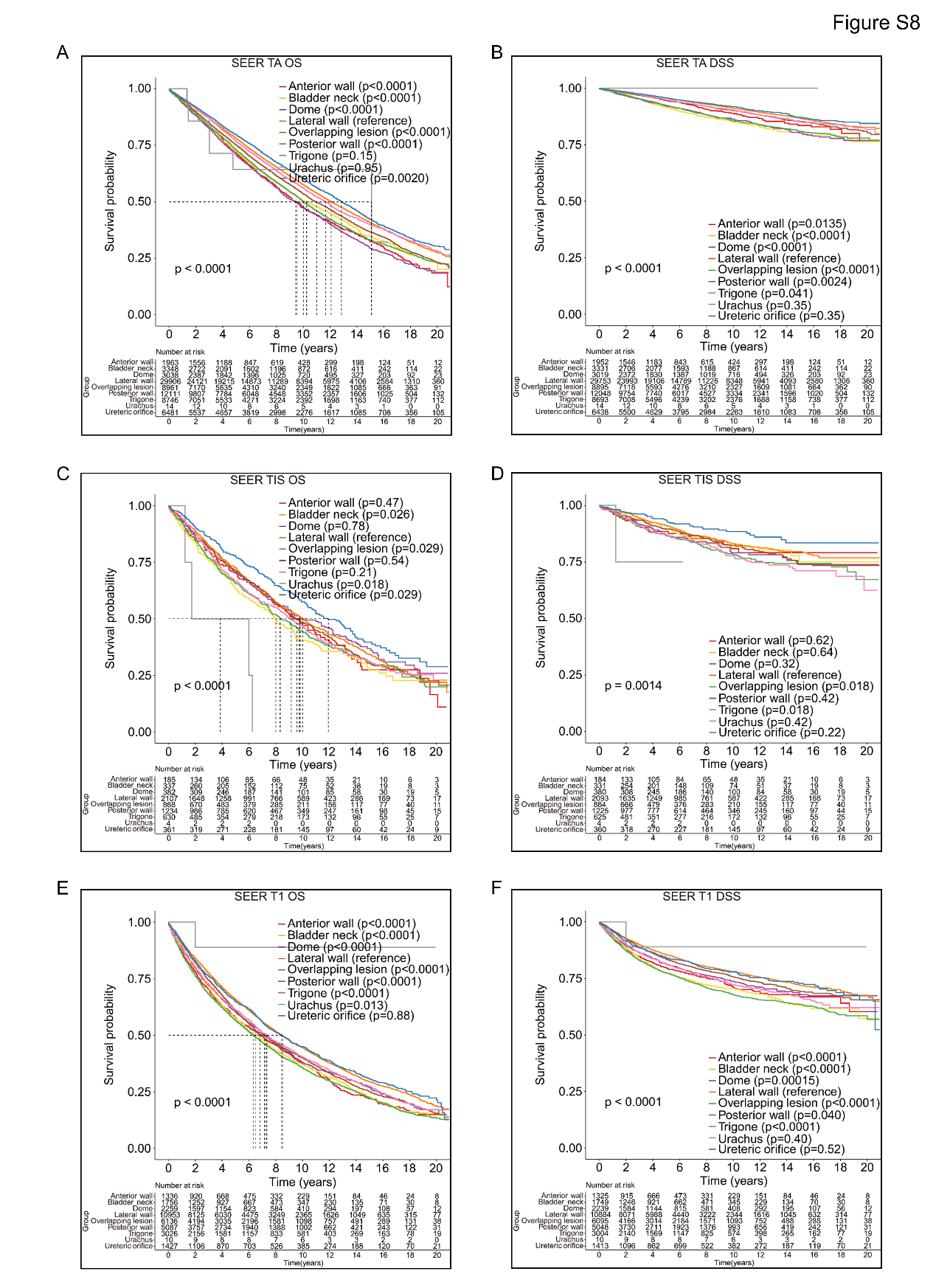


**Figure S9 Comparing the prognosis of patients with tumors in different locations in the Chinese NMIBC cohort, stratified based on Grade.** The overall survival curve and recurrence-free survival curve for Grade-PUNLMP (A-B), Grade-low (C-D) and Grade-high (E-F) patients.


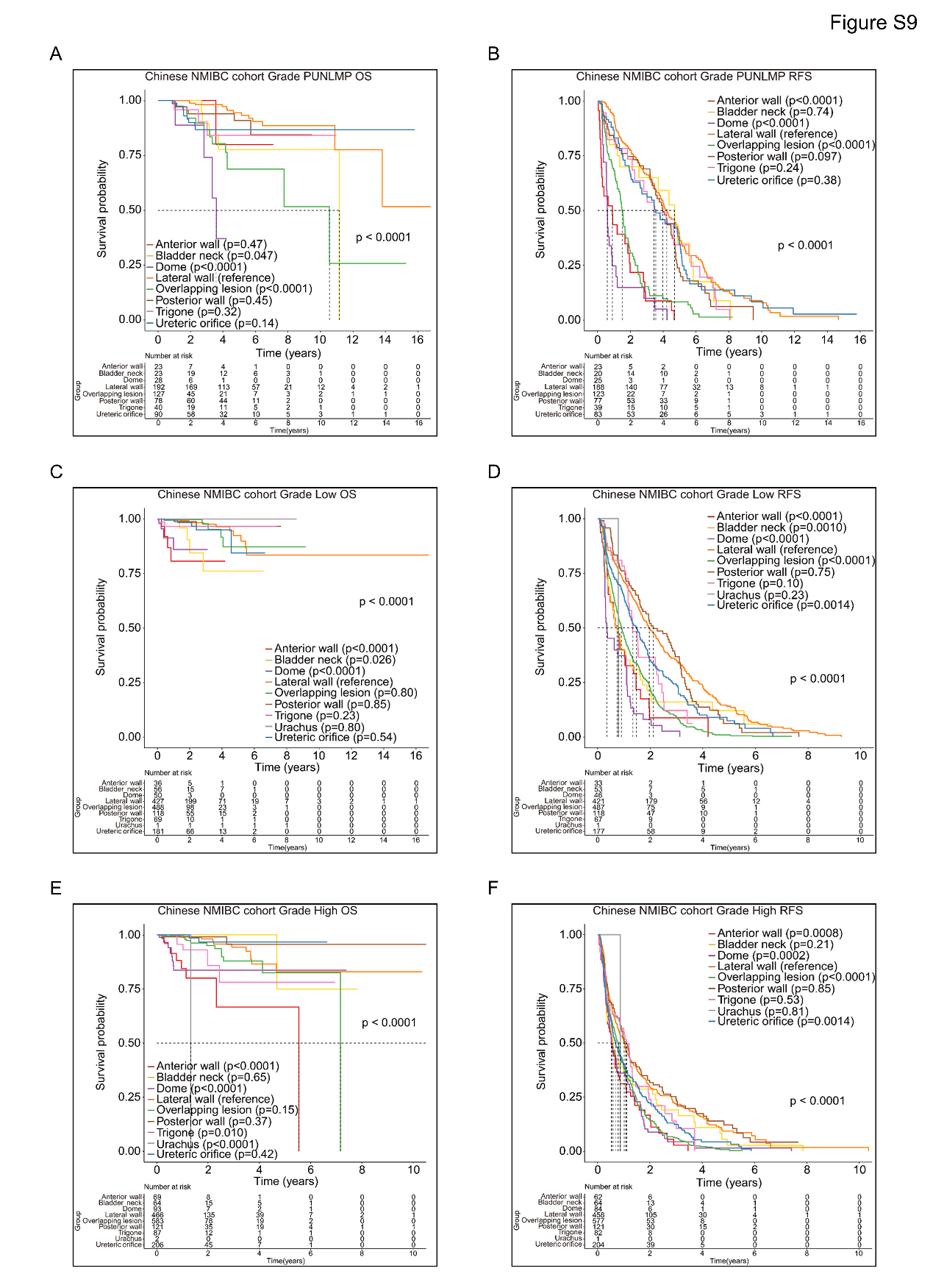


**Figure S10 Comparing the prognosis of patients with tumors in different locations in the SEER cohort, stratified based on Grade.** The overall survival curve and disease-specific survival curve for Grade-low (A-B) and Grade-high (C-D) patients.


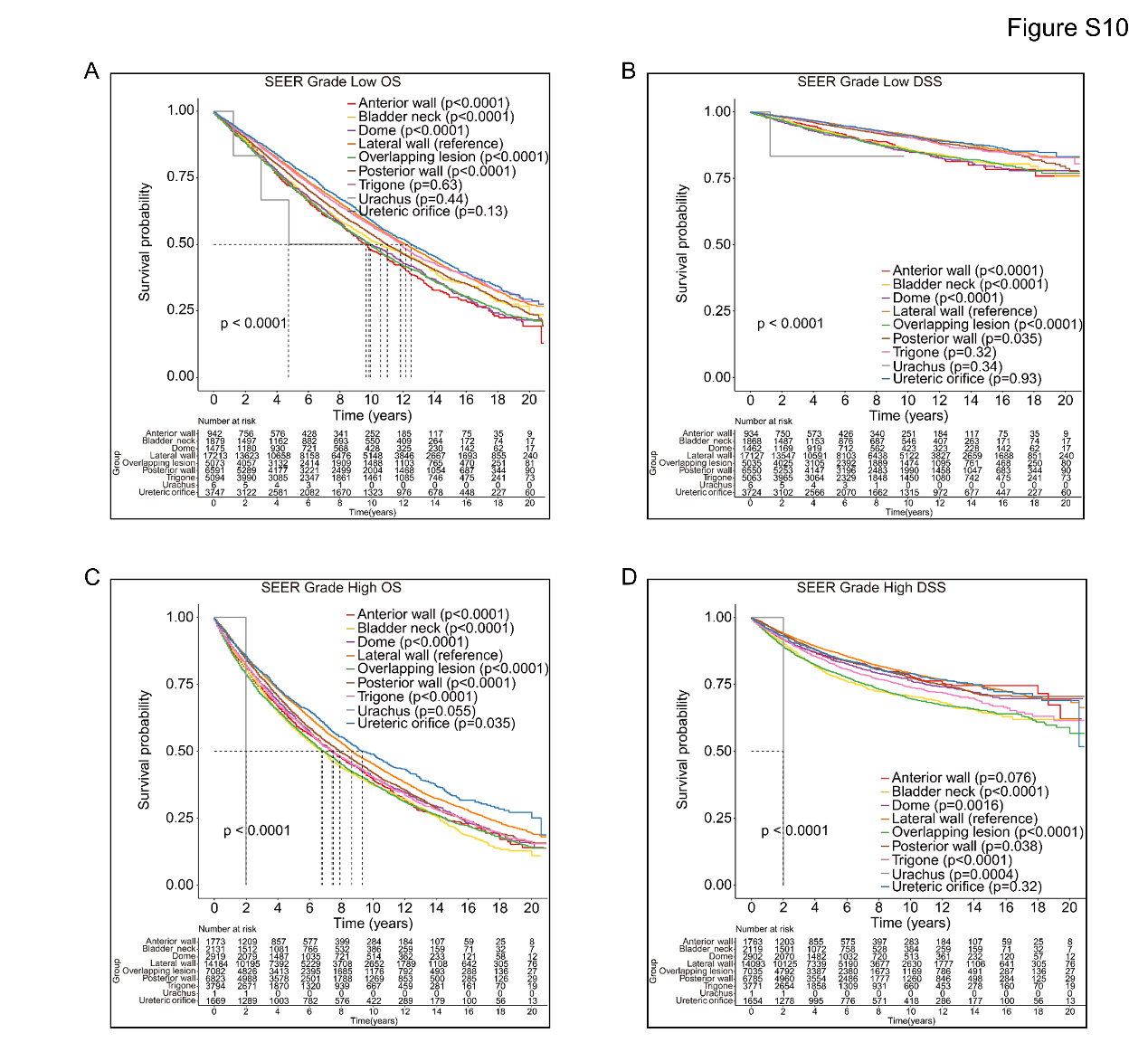


**Figure S11 Comparing the prognosis of patients with tumors in different locations in the Chinese NMIBC cohort, stratified based on number of tumors.** The overall survival curve and recurrence-free survival curve for single tumor (A-B) and multiple tumors (C-D) patients.


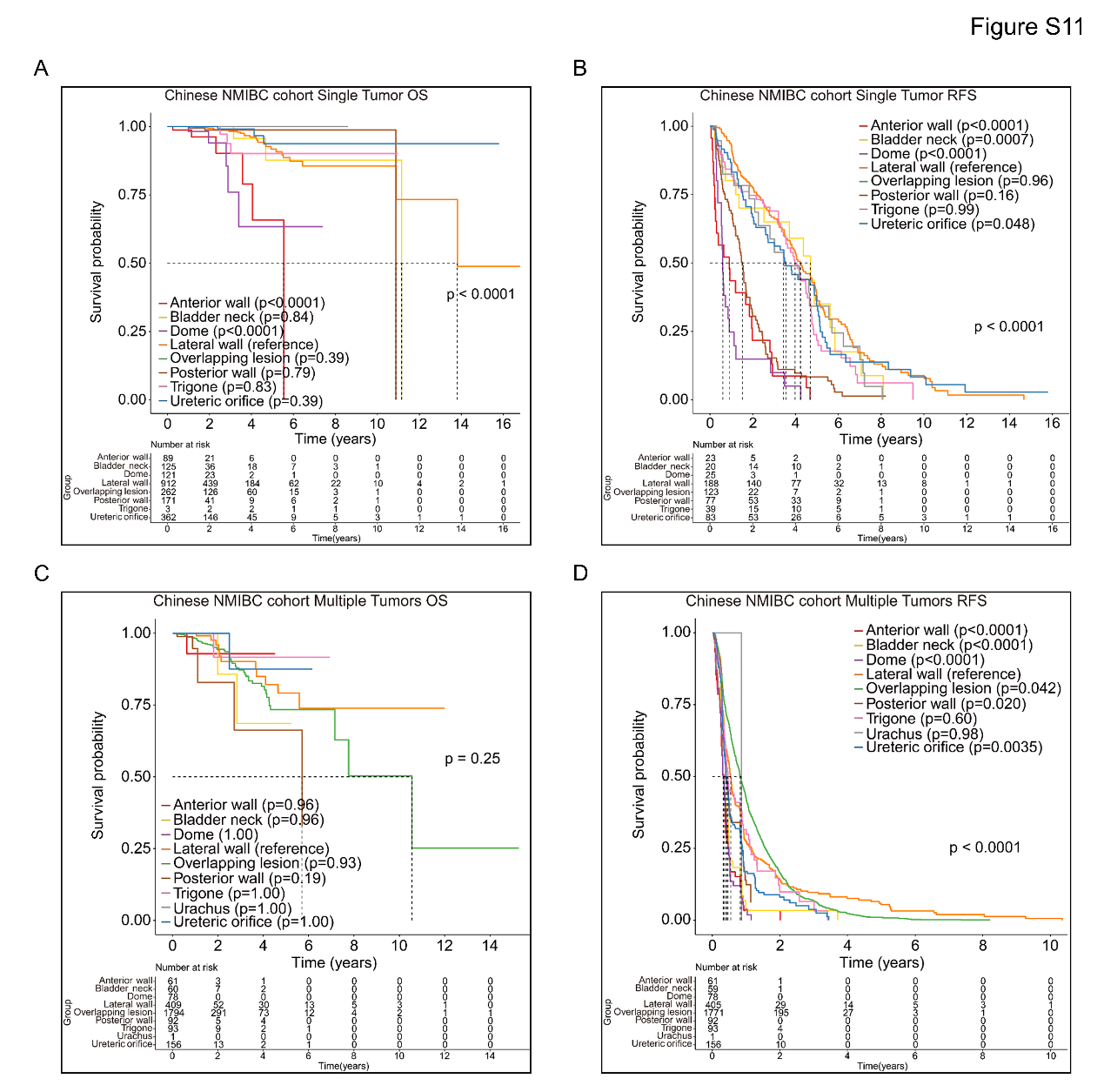


**Figure S12 Comparing the prognosis of patients with tumors in different locations in the SEER cohort, stratified based on Grade.** The overall survival curve and disease-specific survival curve for single tumor (A-B) and multiple tumors (C-D) patients.


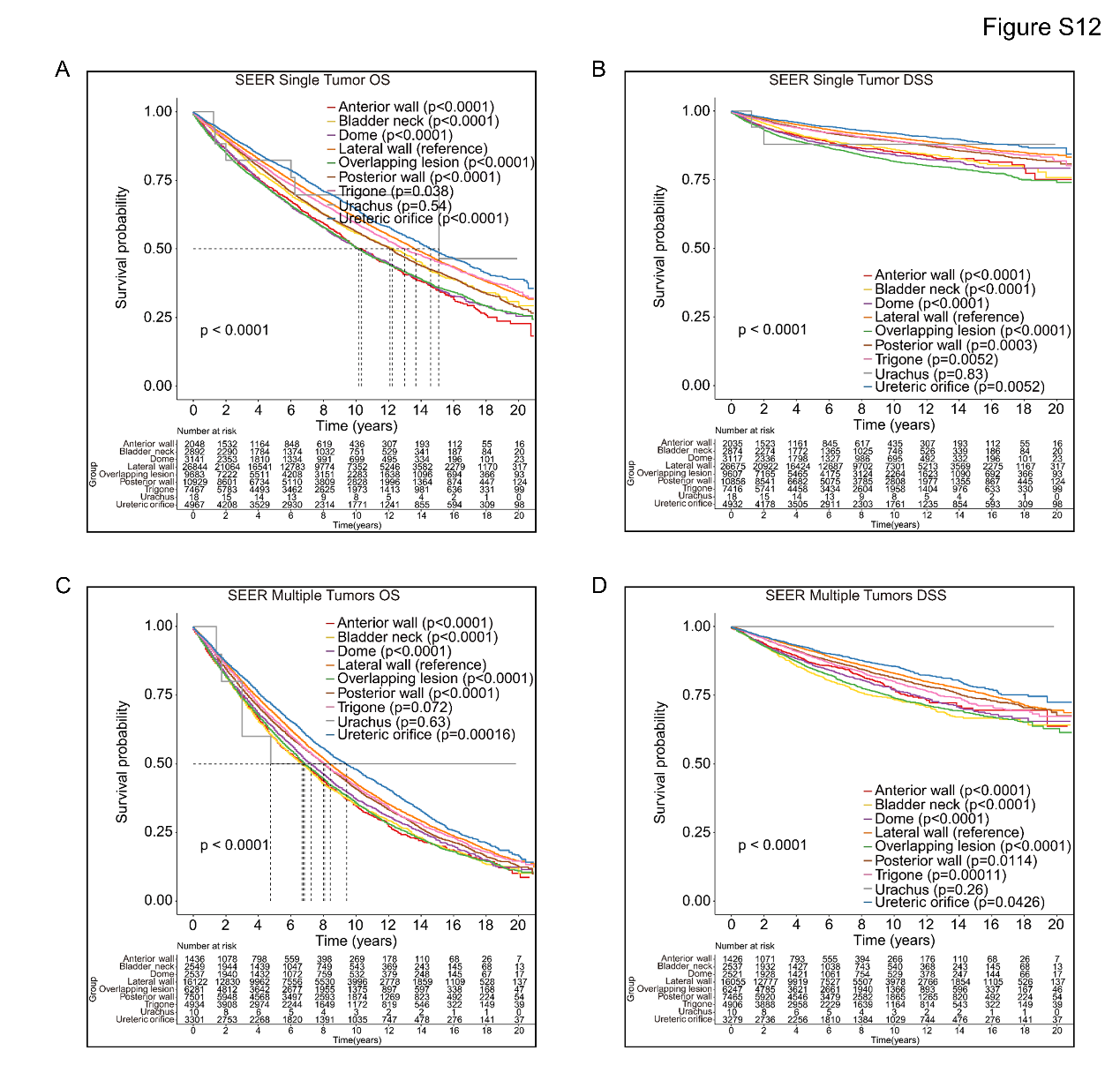


**Figure S13 Comparing the prognosis of patients with tumors in different locations in the SEER cohort, stratified based on tumor size.** The overall survival curve and disease-specific survival curve for tumor size ≤ 20 mm (A-B) and > 20 mm (C-D) patients.


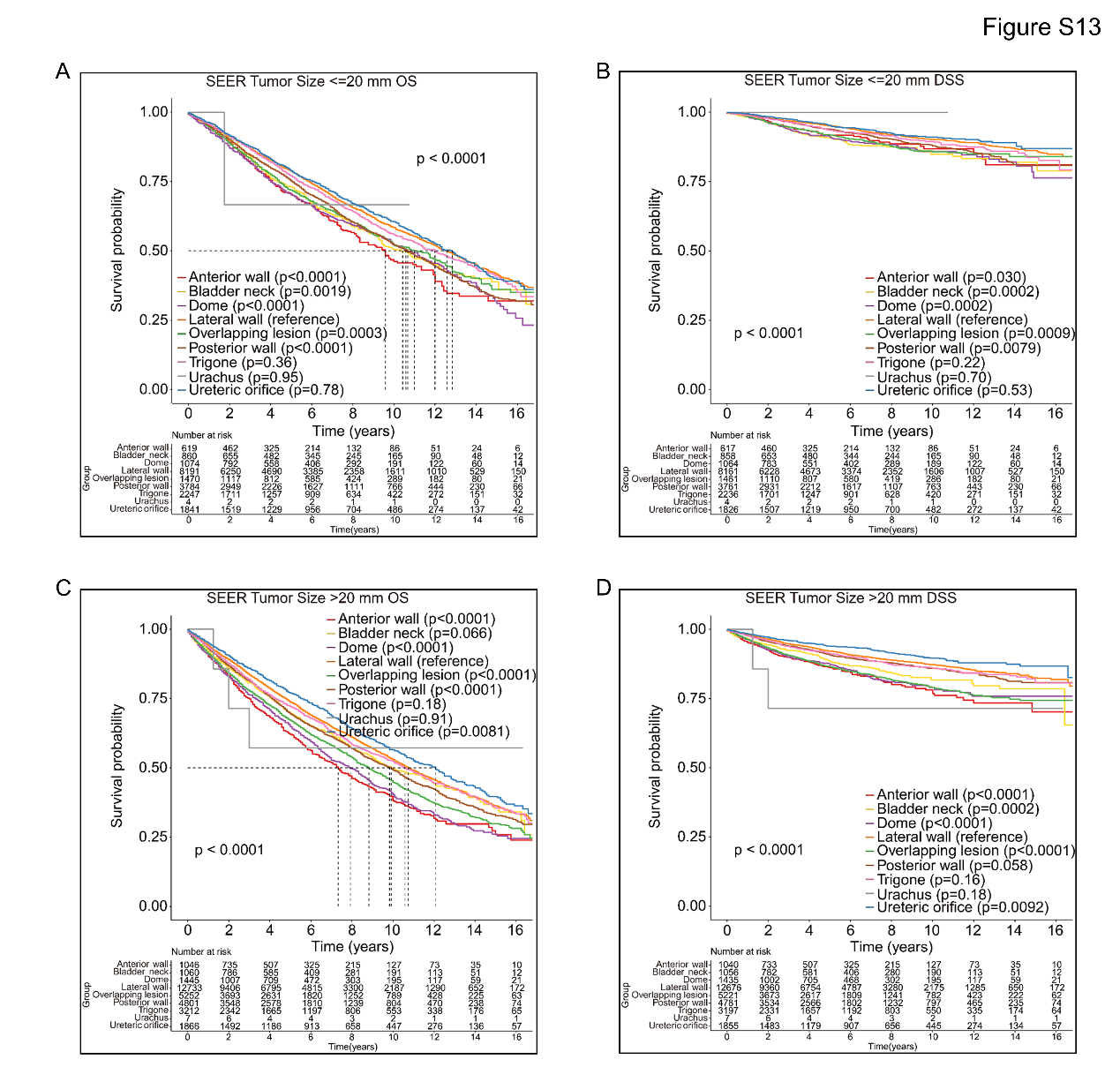


**Figure S14 Univariate COX analysis of the recurrence-free survival of NMIBC patients in the Chinese NMIBC cohort.**


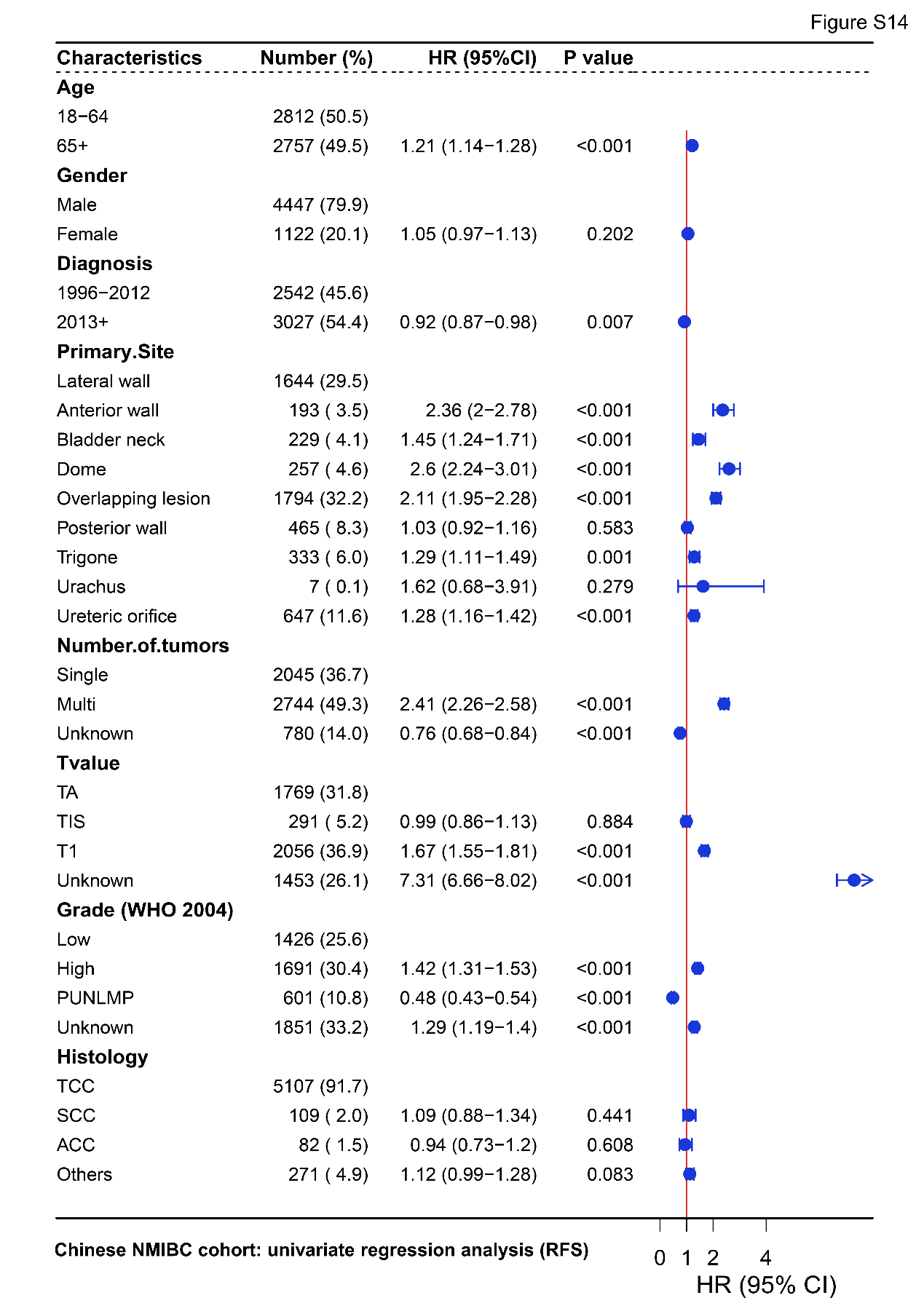


**Figure S15 Univariate COX analysis of the overall survival of NMIBC patients in the SEER cohort.**


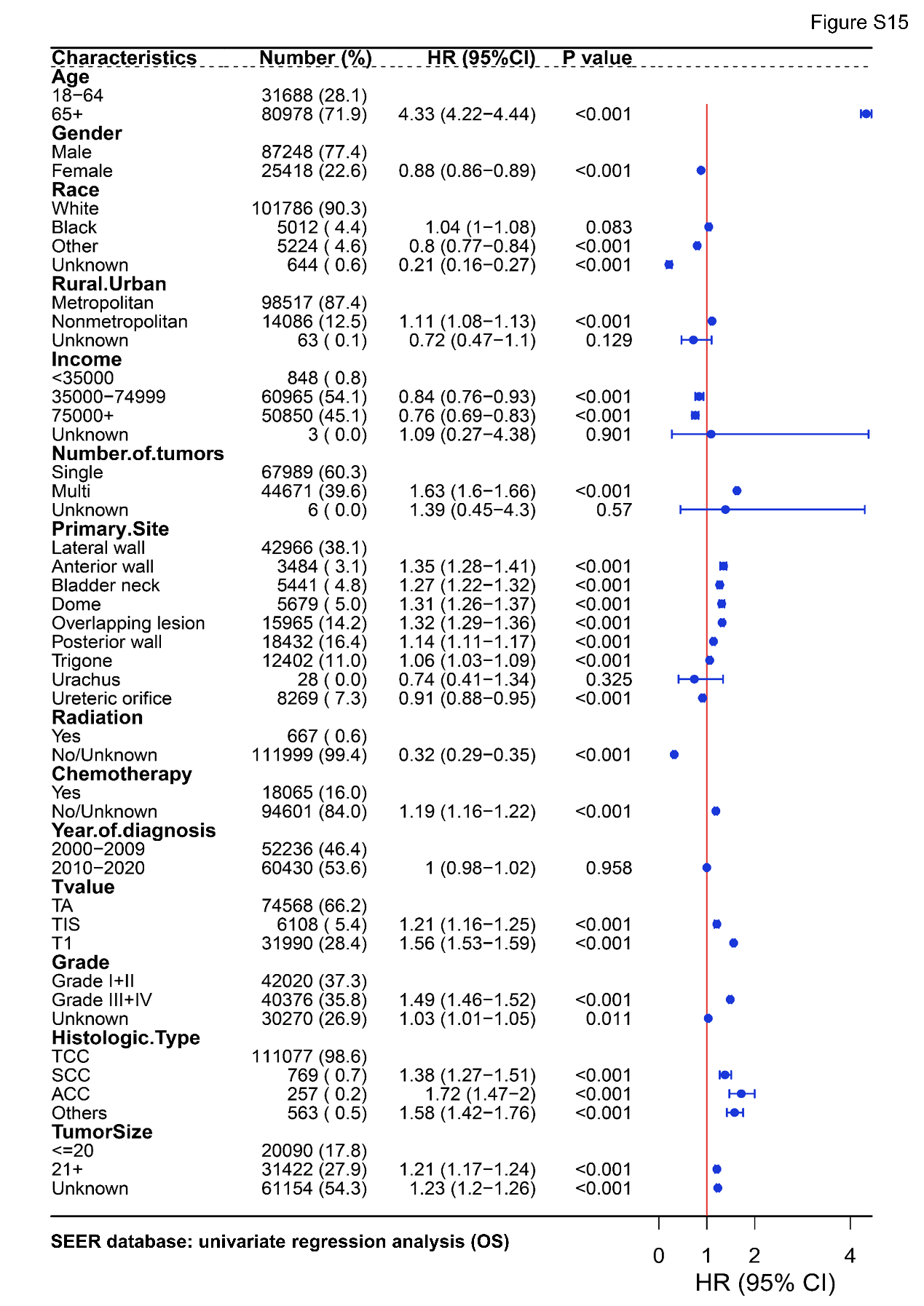

Supplement: Supplementary file 1 [file js9-110-5641-s001.docx]
